# Supplementary material for: Spatial variation and predictors of missing birth preparedness and complication readiness (BPCR) messages in Ethiopia
Source: PLoS One. 2023 Dec 8;18(12):e0295744. doi: 10.1371/journal.pone.0295744 (PMC10707682; doi:10.1371/journal.pone.0295744)
Supplement: S1 File — (PDF) [file pone.0295744.s001.pdf]

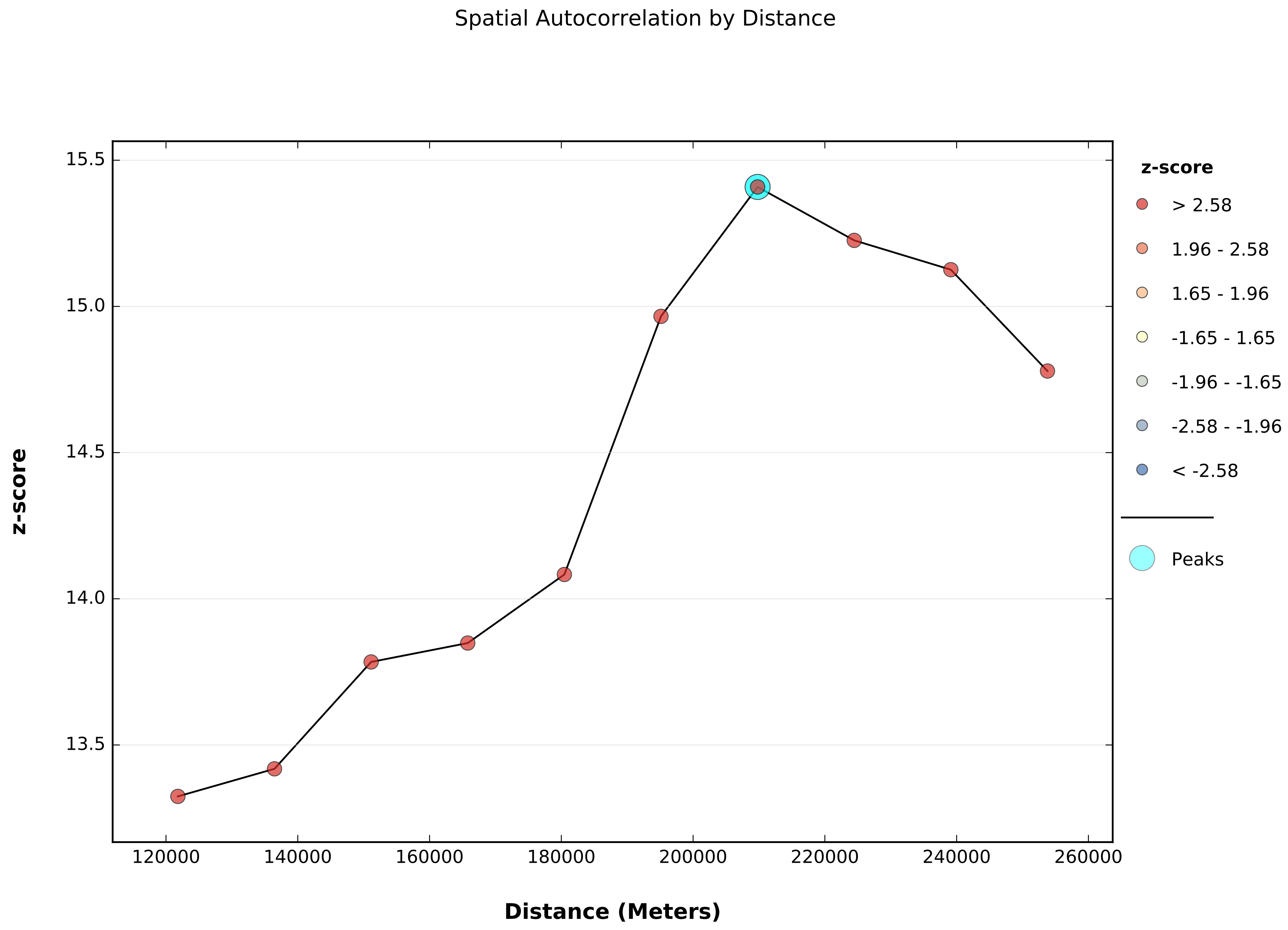

Spatial Autocorrelation by Distance
z-score
15.5
15.0
14.5
14.0
13.5
120000
140000
160000
180000
200000
220000
240000
260000
Distance (Meters)
z-score
> 2.58
1.96 - 2.58
1.65 - 1.96
-1.65 - 1.65
-1.96 - -1.65
-2.58 - -1.96
< -2.58
Peaks

Global Moran's I Summary by Distance

Distance	Moran's Index	Expected Index	Variance	z-score	p-value
121803.00	0.158206	-0.001650	0.000144	13.324095	0.000000
136467.91	0.140250	-0.001650	0.000112	13.418453	0.000000
151132.83	0.132222	-0.001650	0.000094	13.783975	0.000000
165797.74	0.122071	-0.001650	0.000080	13.848596	0.000000
180462.65	0.114826	-0.001650	0.000068	14.083089	0.000000
195127.56	0.113067	-0.001650	0.000059	14.966043	0.000000
209792.48	0.108879	-0.001650	0.000051	15.408585	0.000000
224457.39	0.102304	-0.001650	0.000047	15.225821	0.000000
239122.30	0.096612	-0.001650	0.000042	15.125589	0.000000
253787.21	0.088617	-0.001650	0.000037	14.778965	0.000000

First Peak (Distance; Value): 209792.48; 15.408585

Max Peak (Distance; Value): 209792.48; 15.408585

Distance measured in Meters

Incremental Autocorrelation Parameters

Parameter Name	Input Value
Input Features	Export_Output_3_Project1
Input Field	P_PBCR_NO
Number of Distance Bands	10
Beginning Distance	121803.000000
Distance Increment	14664.912635
Distance Method	EUCLIDEAN
Row Standardization	True
Selection Set	False
