# Supplementary material for: Spatial variation and predictors of missing birth preparedness and complication readiness (BPCR) messages in Ethiopia
Source: PLoS One. 2023 Dec 8;18(12):e0295744. doi: 10.1371/journal.pone.0295744 (PMC10707682; doi:10.1371/journal.pone.0295744)
Supplement: Attachment — Submitted filename: PONE-D-23-28170.pdf.BPCR.pdf [file pone.0295744.s002.pdf]

PLOS ONE

Modeling spatial predictors of missing birth preparedness and complication readiness (BPCR) messages during pregnancy in Ethiopia: a Geographically Weighted Regression

--Manuscript Draft--

Manuscript Number:	PONE-D-23-28170
Article Type:	Research Article
Full Title:	Modeling spatial predictors of missing birth preparedness and complication readiness (BPCR) messages during pregnancy in Ethiopia: a Geographically Weighted Regression
Short Title:	spatial predictors of missing birth preparedness and complication readiness (BPCR) messages
Corresponding Author:	Aklilu Habte, MPH<br>WCU: Wachemo University<br>Hossana, ETHIOPIA
Keywords:	Birth preparedness and complication readiness, spatial analysis, GWR, Ethiopia
Abstract:	Background<br>Birth Preparedness and Complication Readiness (BPCR) message is one of the comprehensive Antenatal Care packages aimed at enhancing maternal and newborn health service uptake by minimizing unnecessary delays. Conducting a spatial analysis could offer a data-driven approach to understanding and addressing the challenges hindering the dissemination and adoption of key BPCR messages. No prior study in Ethiopia has looked at the spatial variation of missing BPCR messages and its predictors. Hence, this study aimed to identify predictors of hotspots for missing BPCR messages at the national level.<br>Methods<br>This study used secondary data from the 2016 Ethiopian Demographic and Health Survey, which was conducted from January 18, 2016 to June 27, 2016. A total weighted sample of 4771 women was considered. Arc-GIS version 10.7 statistical software was used for the spatial analysis. First, spatial autocorrelation (Moran's I) was run to figure out if the spatial variation of missing BPCR messages in Ethiopia was clustered. Hotspot (Getis-OrdGi*) analysis was conducted to identify geographical risk areas. Finally, spatial modelings were carried out via ordinary least squares and geographically weighted regression to identify predictors of hotspots for missing BPCR messages.<br>Results<br>The overall prevalence of missing BPCR messages in Ethiopia was found to be 44.0% (95%CI: 42.6, 45.4%) with a significant spatial variation across regions. Statistically significant hotspots for missing BPCR messages during pregnancy were northern, southern, and western Somali, central Afar, and Gambella regions. Living in the poorest wealth quintile, receiving only one ANC visit, not having access to listening to the radio, having difficulty accessing money to get medical care, not having a mobile phone, and being not covered by health insurance were identified as significant predictors of hot spots for missing BPCR messages.<br>Conclusion<br>The level of missing BPCR messages during pregnancy was found to be high in Ethiopia with a significant spatial variation across regions. Hence, the government and policymakers should tailor strategies for hotspot areas by implementing activities and interventions that increase women's economic capability, health-seeking behavior during pregnancy, and exposure to media by easing access to radio and mobile phones. Furthermore, the regional authorities should focus on strategies that promote universal health coverage through enrolling citizens in health insurance schemes.
Order of Authors:	Aklilu Habte, MPH in Reproductive Health
	Samuel Hailegbreal, MSc in Health Informatics
	Tamirat Melis, MPH in Epidemiology and Biostatstic

Powered by Editorial Manager® and ProduXion Manager® from Aries Systems Corporation

	Dereje Haile, MPH in Reproductive Health
**Additional Information:**	
**Question**	**Response**
**Financial Disclosure**<br><br>Enter a financial disclosure statement that describes the sources of funding for the work included in this submission. Review the submission guidelines for detailed requirements. View published research articles from *PLOS ONE* for specific examples.<br><br>This statement is required for submission and **will appear in the published article** if the submission is accepted. Please make sure it is accurate.<br><br>**Unfunded studies**<br>Enter: *The author(s) received no specific funding for this work.*<br><br>**Funded studies**<br>Enter a statement with the following details:<br>• Initials of the authors who received each award<br>• Grant numbers awarded to each author<br>• The full name of each funder<br>• URL of each funder website<br>• Did the sponsors or funders play any role in the study design, data collection and analysis, decision to publish, or preparation of the manuscript?<br>• **NO** - Include this sentence at the end of your statement: *The funders had no role in study design, data collection and analysis, decision to publish, or preparation of the manuscript.*<br>• **YES** - Specify the role(s) played.<br><br>* typeset	The author(s) received no specific funding for this work.
**Competing Interests**<br><br>Use the instructions below to enter a competing interest statement for this submission. On behalf of all authors, disclose any competing interests that could be perceived to bias this work—acknowledging all financial support and any other relevant financial or non-financial competing interests.	The authors have declared that no competing interests exist.

Powered by Editorial Manager® and ProduXion Manager® from Aries Systems Corporation

This statement is required for submission and **will appear in the published article** if the submission is accepted. Please make sure it is accurate and that any funding sources listed in your Funding Information later in the submission form are also declared in your Financial Disclosure statement.<br><br>View published research articles from *PLOS ONE* for specific examples.<br><br>**NO authors have competing interests**<br><br>Enter: *The authors have declared that no competing interests exist.*<br><br>**Authors with competing interests**<br><br>Enter competing interest details beginning with this statement:<br><br>*I have read the journal's policy and the authors of this manuscript have the following competing interests: [insert competing interests here]*<br><br>* typeset	
**Ethics Statement**<br><br>Enter an ethics statement for this submission. This statement is required if the study involved:<br><br>• Human participants<br>• Human specimens or tissue<br>• Vertebrate animals or cephalopods<br>• Vertebrate embryos or tissues<br>• Field research<br><br>Write "N/A" if the submission does not require an ethics statement.<br><br>General guidance is provided below. Consult the submission guidelines for detailed instructions. **Make sure that all**	Written permission was received from ICF International to access both the DHS and GPS datasets following registration at the DHS Program website(https://dhsprogram.com) with possible justification. The accessed data were only used for the registered research and were not shared with anyone other than the co-authors. The DHS also declared that informed consent was obtained from all subjects and/or their legal guardian during the primary data collection. Furthermore, the Institutional Review Board of Wachemo University College of Medicine and Health Sciences declared that no formal ethics approval was required in this case because it is secondary data, but to ensure the ethical compliance of the research process with the national and international standards.

Powered by Editorial Manager® and ProduXion Manager® from Aries Systems Corporation

**information entered here is included in the Methods section of the manuscript.**<br><br>**Format for specific study types**<br><br>**Human Subject Research (involving human participants and/or tissue)**<br>• Give the name of the institutional review board or ethics committee that approved the study<br>• Include the approval number and/or a statement indicating approval of this research<br>• Indicate the form of consent obtained (written/oral) or the reason that consent was not obtained (e.g. the data were analyzed anonymously)<br><br>**Animal Research (involving vertebrate animals, embryos or tissues)**<br>• Provide the name of the Institutional Animal Care and Use Committee (IACUC) or other relevant ethics board that reviewed the study protocol, and indicate whether they approved this research or granted a formal waiver of ethical approval<br>• Include an approval number if one was obtained<br>• If the study involved *non-human primates*, add *additional details* about animal welfare and steps taken to ameliorate suffering<br>• If anesthesia, euthanasia, or any kind of animal sacrifice is part of the study, include briefly which substances and/or methods were applied<br><br>**Field Research**<br><br>Include the following details if this study involves the collection of plant, animal, or other materials from a natural setting:<br>• Field permit number<br>• Name of the institution or relevant body that granted permission	
**Data Availability**<br><br>Authors are required to make all data underlying the findings described fully available, without restriction, and from the time of publication. PLOS allows rare exceptions to address legal and ethical	No - some restrictions will apply<br><br>Can you please mention what sorts of restriction?

concerns. See the PLOS Data Policy and FAQ for detailed information.<br><br>A Data Availability Statement describing where the data can be found is required at submission. Your answers to this question constitute the Data Availability Statement and **will be published in the article**, if accepted.<br><br>**Important:** Stating 'data available on request from the author' is not sufficient. If your data are only available upon request, select 'No' for the first question and explain your exceptional situation in the text box.<br><br>Do the authors confirm that all data underlying the findings described in their manuscript are fully available without restriction?	
**Describe where the data may be found in full sentences. If you are copying our sample text, replace any instances of XXX with the appropriate details.**<br><br>• If the data are **held or will be held in a public repository**, include URLs, accession numbers or DOIs. If this information will only be available after acceptance, indicate this by ticking the box below. For example: *All XXX files are available from the XXX database (accession number(s) XXX, XXX.*).<br>• If the data are all contained **within the manuscript and/or Supporting Information files**, enter the following: *All relevant data are within the manuscript and its Supporting Information files.*<br>• If neither of these applies but you are able to provide **details of access elsewhere**, with or without limitations, please do so. For example:<br><br>*Data cannot be shared publicly because of [XXX]. Data are available from the XXX Institutional Data Access / Ethics Committee (contact via XXX) for researchers who meet the criteria for access to confidential data.*	The data for this study were obtained from the DHS program with a reasonable request. Thus, the one who needs the data supporting the findings of this study can get it in anonymized form from the DHS website at https://www.dhsprogram.com upon reasonable request in the same manner as the authors did.

The data underlying the results presented in the study are available from (include the name of the third party and contact information or URL).<br>• This text is appropriate if the data are owned by a third party and authors do not have permission to share the data.<br><br>* typeset	
Additional data availability information:	

Powered by Editorial Manager® and ProduXion Manager® from Aries Systems Corporation

Manuscript Click here to access/download;Manuscript;Manuscript.docx

Modeling spatial predictors of missing birth preparedness and complication readiness (BPCR) messages during pregnancy in Ethiopia: a Geographically Weighted Regression

Aklilu Habte[1], Samuel Hailegebireal[2], Tamirat Melis[3], Dereje Haile[4]

[1] School of Public Health, College of Medicine and Health Sciences, Wachemo University, Hosanna, Ethiopia

[2] College of Medicine and Health Sciences, School of Public Health, Department of Health Informatics, Wachemo University, Hosaena, Ethiopia

[3] Department of Public Health, College of Medicine and Health Sciences, Wolkite University, Wolkite, Ethiopia

[4]Department of Reproductive Health, College of Medicine and Health Sciences, School of Public Health, Wolaita Sodo University, Sodo, Ethiopia

[*]Corresponding author: akliluhabte57@gmail.com

Abstract

Background

Birth Preparedness and Complication Readiness (BPCR) message is one of the comprehensive Antenatal Care packages aimed at enhancing maternal and newborn health service uptake by minimizing unnecessary delays. Conducting a spatial analysis could offer a data-driven approach to understanding and addressing the challenges hindering the dissemination and adoption of key BPCR messages. No prior study in Ethiopia has looked at the spatial variation of missing BPCR messages and its predictors. Hence, this study aimed to identify predictors of hotspots for missing BPCR messages at the national level.

Methods

This study used secondary data from the 2016 Ethiopian Demographic and Health Survey, which was conducted from January 18, 2016 to June 27, 2016. A total weighted sample of 4771 women was considered. Arc-GIS version 10.7 statistical software was used for the spatial analysis. First, spatial autocorrelation (Moran's I) was run to figure out if the spatial variation of missing BPCR messages in Ethiopia was clustered. Hotspot (Getis-OrdGi*) analysis was conducted to identify geographical risk areas. Finally, spatial modelings were carried out via ordinary least squares and geographically weighted regression to identify predictors of hotspots for missing BPCR messages.

Results

The overall prevalence of missing BPCR messages in Ethiopia was found to be 44.0% (95%CI: 42.6, 45.4%) with a significant spatial variation across regions. Statistically significant hotspots for missing BPCR messages during pregnancy were northern, southern, and western Somali, central Afar, and Gambella regions. Living in the poorest wealth quintile, receiving only one ANC visit, not having access to listening to the radio, having difficulty accessing money to get medical care, not having a mobile phone, and being not covered by health insurance were identified as significant predictors of hot spots for missing BPCR messages.

Conclusion

The level of missing BPCR messages during pregnancy was found to be high in Ethiopia with a significant spatial variation across regions. Hence, the government and policymakers should tailor strategies for hotspot areas by implementing activities and interventions that increase women's economic capability, health-seeking behavior during pregnancy, and exposure to media by easing access to radio and mobile phones. Furthermore, the regional authorities

should focus on strategies that promote universal health coverage through enrolling citizens in health insurance schemes.

Introduction

Please confirm the full form of LMICs)

Despite a 34% decline in maternal mortality ratio (MMR) between 2000 and 2020, it remains unacceptably high in 2020, with around 287,000 women dying during and after pregnancy and childbirth[1, 2]. Almost 95% of all maternal deaths occurred in low and lower-middle-income countries(LMICs) in 2020, and most could have been prevented[3]. Sub-Saharan Africa (SSA) and Southern Asia accounted for approximately 70 percent (202,000) of the estimated global maternal deaths in 2020[1, 2]. Although Ethiopia has lowered maternal and child mortality by half in the last 15 years, from an MMR of 871 to 412 per 100,000 live births and a child mortality rate of 67 per 1,000 are still far too high[4, 5].

Preventable direct obstetric causes such as severe bleeding (usually the postpartum one), puerperal infections, high blood pressure during pregnancy (pre-eclampsia and eclampsia), delivery-related complications (obstructed labor and ruptured uterus), and unsafe abortion account for about 75% of all maternal deaths[1]. Delays in preparing for and responding to those issues during pregnancy and childbirth by pregnant women, family, and health care providers have been identified as the main hurdle in the reduction of maternal death and illness in SSA[6]. In this respect, the Birth preparedness and complication readiness (BPCR) package is a key instrument for addressing such delays in Sub-Saharan Africa (SSA), especially Ethiopia, where obstetric services are often limited and/or underutilized [7].

Birth preparedness and complication readiness (BPCR) is one of the WHO recommendations for newborn and maternal health promotion activities that are delivered and executed through an antenatal care(ANC) program[8]. BPCR is defined as a comprehensive program strategy to increase the adoption and effectiveness of essential maternal and newborn health care by reducing three delays in accessing basic maternal health services (delays in deciding, reaching, and receiving care)[9]. It encourages the timely utilization of skilled maternal and neonatal care, on the assumption that planning for childbirth and being prepared for complications lowers lags in receiving those basic care[8, 10]. It has a set of indicators that were intended to be implemented by pregnant women, families(husband/partner), communities, health facilities, and health care providers[11]. Those items of care that are intended to be implemented by all pregnant women are: deciding on a birthplace, maintaining the necessary supplies for childbirth, figuring out or establishing emergency transportation, setting aside

money or an emergency fund, identifying blood donor/s, determining companions during labor and childbirth which are vital in easing access to care [11, 12].

Despite having a considerable impact on maternal mortality reduction, BPCR messages were not widely implemented in Ethiopia. A systematic review and meta-analysis conducted in Ethiopia revealed that BPCR uptake was 25.2%[12]. On the other hand, a recent study from nationally representative data showed that more than half (56.02%) of women received at least one BPCR message [10]. Small-scale studies conducted in the country showed that there was a positive association between the receipt of BPCR messages and educational status, occupation, being in a higher wealth quintiles, receipt of ANC visits, previous history of health facility delivery, history of adverse birth outcomes, number of living children (parity), knowledge of key danger signs during pregnancy and postpartum, and male partner involvement in BPCR plan [13-18].

Despite numerous studies on the uptake of BPCR messages across the country, no existing study has investigated the spatial distribution that shows regions with poor uptake of BPCR messages. Hence, the current study carried out spatial analytic techniques (a hot spot and a geographically weighted regression (GWR) analysis). First, a hotspot analysis was carried out to identify the regions and areas with low BPCR message adoption, and the study will next delve into identifying potential variables that lead to regional differences in poor service uptake by employing a GWR. Conducting a hotspot analysis could offer a data-driven approach to understanding and addressing the challenges hindering the dissemination and adoption of key BPCR messages. By identifying specific geographic regions with low BPCR message adoption, resources and interventions can be allocated to areas of highest need and groups that are especially vulnerable to maternal complications of pregnancy. The findings from the current study will allow program planners, policymakers, and healthcare providers to customize their efforts to address the factors behind poor service uptake in each hotspot, thereby increasing the likelihood of successful intervention. By targeting interventions in hotspots with low BPCR message uptake, maternal and neonatal health outcomes can be significantly improved via working on safer pregnancies, and timely access to healthcare services.

please do segregate this part

Methods and materials

Study setting, data source, and study period

The current study was based on the report of the 2016 Ethiopian Demographic and Health Survey (EDHS) data, which was the fourth one. The survey was conducted by the Central

Statistics Agency (CSA) from January 18, 2016, to June 27, 2016, with the primary objective of providing up-to-date estimates of key demographic and health indicators[5]. It also provides an in-depth overview of the population, and maternal, and child health issues in the country. Ethiopia is located in the Horn of Africa (3^0–15^0 N latitude and 33^0–48^0 E longitude). The country is divided into nine administrative regions (Tigray, Afar, Amhara, Oromia, Somali, Benishangul-Gumuz, Southern Nation Nationality and People's Region (SNNPR), Gambella, and Harari) and two self-administrative cities (Addis Ababa and Dire Dawa).

Please do write this section again as the study population are not clearly mention , please give more background of the study population . Also, What do you mean by preceding over here, can you please make it more clear?What is source popualtion and their purpose? Please clearify the inclusion and exclusion criteria in a clear way.

The population of the study

All women aged 15 to 49 who were either permanent inhabitants or people who stayed in the selected houses preceding the survey were eligible for the study. The source populations were women who gave birth within the five years preceding the survey, whereas the study populations were women who had complete information on the uptake of BPCR messages during their ANC visit. The EDHS has numerous datasets, for males, women, children, birth, and households. The women's (IR file) was used for the current study, and a total weighted sample of 4,771 women was considered in the analysis.

Sampling procedures and data collection tool

The Ethiopia Population and Housing Census (PHC), carried out in 2007 by the Ethiopia CSA, served as the sampling frame for the 2016 EDHS. The samples were chosen using a stratified, two-stage cluster sampling procedure in which data were hierarchical. In the first stage, 645 enumeration areas (EAs) were selected (202 in urban and 443 in rural areas), with a probability proportional to EA size and independent selection in each sampling stratum. In each of the selected EAs, a household listing operation was carried out, and the consequent lists of households served as a sampling frame for the selection of households in the second stage. In the second stage, a fixed number of 28 houses per cluster were chosen with equal probability from the sample frame[5].

How the validity and reliabilty of the questanaire was confirmed?

The Woman's Questionnaire, which includes socio-demographic and economic information, obstetric characteristics, and maternal health service utilization, was used to collect data from all eligible women aged 15 to 49. The location data (geographic coordinates) of each survey cluster were collected using Global Positioning System (GPS) receivers. Each cluster's GPS reading was taken in the center. To protect respondents' privacy, GPS latitude/longitude positions for all survey groups were randomly displaced. The highest displacement for urban clusters was two kilometers (km), whereas, for 99% of rural clusters, it was five kilometers (km). The remaining 1% of rural clusters have been relocated up to a distance of ten km[19].

Measurement of variables of the study

The outcome variable was missing BPCR messages and was measured based on the six key WHO-recommended messages received by a mother during her last pregnancy. The key messages were about deciding the place of birth, collecting the necessary supplies for childbirth, arranging emergency transportation, setting money aside for emergency conditions, identifying companions during labor and childbirth, and securing potential blood donors. Information on these six key BPCR messages was derived from the response to the question: Please,Confirm the question again Were you told about your Place of birth? Were you told about supplies needed for birth? Were you told about emergency transportation?... The responses were recorded as Yes (=1) or No (=0). A single woman may be advised about any of the six messages many times throughout the same pregnancy, but each response was recorded as a single message. Based on the responses, a composite index of BPCR was created, which had a minimum and maximum value of 0 and 6 respectively. The women with '0' values were regarded as 'missing BPCR messages,' whereas those with one or more messages were considered to have received BPCR[10, 20].

Explanatory variables

Potential determinants of BPCR were extracted from the data set after reviewing related and current literature [10-12, 18, 21](Table 1).

Table 1: Potential determinants extracted from the EDHS 2016 report for studying predictors of not receiving BPCR messages

Variables	Description	Category
Age	The respondent's age, expressed in years, at the time of the survey.	1. 15–19,<br>2. 20–34<br>3. 35-49
Marital status	Percentage of women according to the current status of marriage or cohabitation.	1. Married<br>2. Never married<br>3. Others
Women educational attainment	Percent distribution of women ages 15-49 by the highest level of schooling attended or completed.	1. No education<br>2. Primary<br>3. Secondary<br>4. Higher
Residence	The area where respondents lived when the survey was conducted.	1. Urban<br>2. Rural
Family size	Number of household members at the time of data collection	1. ≤5<br>2. >5
Sex of head of Household	Percent distribution of households by sex of head of household	1. Female<br>2. Male
Wealth index	Calculated using easy-to-collect data on a household's ownership of selected assets, such as televisions and bicycles; materials used for housing construction; and types of water access and sanitation facilities.	1. Richest<br>2. Richer<br>3. Middle<br>4. Poorer

		5. Poorest
Parity	The number of living children the woman had at the time of the survey	1. Nulliparous<br>2. Primiparous<br>3. Multiparous<br>4. Grand multiparous
Pregnancy status during last childbirth	Percentage of births to women aged 15-49 in the five years preceding the survey, including current pregnancies, by birth planning status - (i) wanted then, (ii) wanted later, or (iii) not wanted at all.	1. Unwanted (*ii& iii)*<br>2. Wanted (*i*)
Media exposure	The number of women aged 15 to 49 who are exposed to specific media at various frequencies, such as reading a newspaper, watching television, and listening to the radio.	1. Not at all<br>2. Less than once a week<br>3. At least once a week
Autonomy in decision-making [a]	The total number of currently married women aged 15 to 49 who make decisions about their own health care, big household purchases, and visits to family or relatives.	1. Low<br>2. Medium<br>3. high

Autonomy in decision-making was measured using replies to questions about who makes the ultimate decision for the family on big property purchases, visits to relatives, and health care. (i) respondent alone, (ii) respondent and husband/partner, (iii) husband/partner alone, (iv) someone else, and (v) others were the response categories. For each question, replies (i) or (ii) earned a score of 1, indicating good decision-making capacity, while the remaining responses were assigned a value of 0, indicating limited capacity. The responses on each of the three aspects were added together to get a total score ranging from 0 to 3. Finally, a composite score had been divided into two unique groups: low and high for "0 to 2" and "3" scores, respectively[22, 23].

Data management and statistical analysis

STATA version 14.1 software was used for data extraction, cleaning, coding, and analysis, while ArcGIS 10.7 was used for spatial analysis. The data were weighted to minimize under- or over-representation in the surveys due to strata-specific selection. The weighting procedure was thoroughly discussed in the 2016 EDHS report[5]. The weighted proportions of a response and explanatory variables have been estimated in STATA and exported to Microsoft Excel 2016 before being loaded into ArcGIS 10.7 for further analysis. To describe the background characteristics of respondents, descriptive statistics such as frequency and percentage were used.

Spatial analysis

Spatial autocorrelation

To figure out if the spatial distribution of not receiving BPCR messages in Ethiopia was dispersed, clustered, or randomly distributed, the global spatial autocorrelation (Global Moran's I) was carried out[24]. The Global Moran's I statistic is used to quantify spatial

autocorrelation by taking the whole data set and producing a single output value ranging from -1 to +1. Moran's I value at -1, +1, and 0 indicates the likelihood of missing BPCR messages being dispersed, clustered, and randomly distributed, respectively. The Z-score determines the statistically significant difference in clustering, and the p-value determines the significance. Accordingly, Moran's, I value was statistically significant ($p<0.05$), showing the presence of spatial autocorrelation and it showed the need for hot spot analysis[25].

Incremental spatial autocorrelation

The peak distance from the spatial incremental autocorrelation model identifies the place where the spatial dependency of missing BPCR messages was most evident and that highest peak distance value has been selected as the threshold distance for hotspot analysis**(S1 File)**[26].

Hot spot analysis (Gettis-Ord Gi* statistics)

The local spatial analysis was performed using Getis-Ord Gi* statistics to identify important hot spot and cold spot areas for missing BPCR messages. The hotspot analysis compares the local mean rate (the rates for a cluster and its nearest neighbouring clusters) to the global mean rate (the rates for all clusters). The z-score was determined to confirm the statistical significance of clustering, and the p-value was calculated to establish the significance at a p-value<0.05 with 95% CI. Cold spots were announced if the z-score is less than -1.96, and hotspot locations were declared if the z-score was greater than +1.96 [24, 27-29]. In addition, Incremental Spatial Autocorrelation was examined to determine a suitable threshold for finding spatial processes that favour clustering[30].

Spatial interpolation

Based on reported or observed values from selected enumeration areas (EAs), spatial interpolation was used to anticipate the proportion of missing BPCR messages in unsampled EAs. For doing this, the standard Kriging interpolation method was used instead of other interpolation approaches since it is an optimal interpolator with a minimum mean error (ME) and root mean square error (RMSE)[31, 32].

Spatial regression analysis.

Spatial regression incorporates both global (ordinary list squares) and local(geographically weighted regression) analysis approaches[33, 34].

Ordinary list squares (OLS) regression

The Ordinary Least Squares (OLS) model is a global statistical model used to evaluate and explain the relationship between outcome and explanatory variables[35]. It served as a tool for diagnosis as well as to pick the best predictors for the Geographically Weighted Regression (GWR) model. Findings from OLS regression are only reliable if the regression model meets

all of the assumptions necessary by this method. In a properly fitted OLS model, coefficients of predictors should be statistically significant and have either a positive or negative sign. Furthermore, there should be no multicollinearity among explanatory variables [34, 36]. Multicollinearity was examined using the Variance Inflation Factor (VIF), and those variables with VIF > 10 were deemed as multicollinear (redundant) and were removed from the model. Furthermore, the Koenker Bp statistic had been utilized to determine whether the model could be used to do a spatially weighted regression analysis. The Koenker statistics were significant (p-value=0.0198) in the current study, and GWR analysis was necessary to analyze the distribution of relevant predictors.

Geographically weighted regression (GWR)

It is evident that a strong predictor in one cluster is not always a strong predictor in another. The application of GWR can identify this form of cluster variation (non-stationarity) of predictors[37]. Unlike OLS which fits a single linear regression equation to all of the data of all features (in this instance, the clusters), GWR uses data from neighboring features, so the GWR coefficient has distinct values for each cluster[38]. The six checks indicated for spatial regression analysis were also carried out. [37]. Variables with p-values less than 0.05 are chosen and discussed based on their coefficients. Finally, the model with the lowest AICc score and the highest adjusted R-squared value was determined to be the ideal fit for the data.

Ethical consideration and consent to participate

Written permission was received from ICF International to access both the DHS and GPS datasets following registration at the DHS Program website(https://dhsprogram.com) with possible justification. The accessed data were only used for the registered research and were not shared with anyone other than the co-authors. The DHS also declared that informed consent was obtained from all subjects and/or their legal guardian during the primary data collection. Furthermore, the Institutional Review Board of Wachemo University College of Medicine and Health Sciences declared that no formal ethics approval was required in this case because it is secondary data, but to ensure the ethical compliance of the research process with the national and international standards.

Results

Socio-demographic characteristics of respondents

A total weighted sample of 4771 women was considered in the current study. The mean (±SD) age of women was 28.79(±6.61) years, with more than half (52.3%) of them belonging to the age group 25-34 years. One-third (33.7%) of study participants were from the Oromia region.

The vast majority of respondents (81.7%) were from rural areas of the country, and more than half (54.1%) had no formal education. The proportion of women who missed BPCR messages was higher among those living in the poorest wealth quintile (51.0%), and received only one ANC visit(65.8%). Furthermore, 44.7% of women living in rural areas did not receive BPCR messages (Table 2).

Table 2: The weighted proportion of receipt of BPCR messages across selected characteristics of the respondents in Ethiopia, EDHS 2016

Variable categories	Total [Weighted frequency (%)]	Receipt of BPCR message		
		Yes (%)	No (%)	Test statistics
**Age**				
15-24	1,236(25.9)	670(54.2)	566(45.8)	X2= 9.12 p=0.071
25-34	2,496(52.3)	1,377(55.2)	1,119(44.8)	
35-49	1,039(21.8)	625(60.1)	414(39.9)	
**Marital status**				
Married and cohabiting	4,481(93.8)	2,507(56.0)	1,974 (44.0)	X2= 2.02 p= 0.089
Not in marital relationship	291(6.2)	165(56.7)	126(43)	
**Regions**				
Tigray	486(10.2)	375(77.2)	111(22.8)	X2= 423.70 p<0.001
Afar	37(0.8)	8(21.9)	29(78.1)	
Amhara	1,104(23.1)	727(65.9)	377(34.1)	
Oromia	1,607(33.7)	744(46.3)	863(53.7)	
Somali	11(2.5)	39(32.8)	79(67.2)	
Benishangul	56(1.2)	27(48.9)	29(51.1)	
SNNPR	1,115(23.4)	604(54.2)	511(45.8)	
Gambella	15(0.3)	6(42.9)	9(57.1)	
Harari	13(0.3)	8(60.8)	5(39.2)	
Addis Ababa	192(4.0)	121(62.9)	71(37.1)	
Diredawa	29(0.6)	13(45.6)	16(54.4)	
**Residence**				
Urban	875(18.3)	517(59.1)	358 (40.9)	X2= 42.13 P=0.011
Rural	3,896 (81.7)	2,155(55.3)	1,741(44.7)	
**Wealth index combined**				
Poorest	794(16.7)	389(49.0)	405(51.0)	X2= 94.16 P<0.001
Poorer	935(19.6)	467(49.9)	468(50.1)	
Middle	996(20.9)	565(56.7)	431(43.3)	
Richer	967(20.2)	568(58.8)	398(41.2)	
Richest	1,079(22.6)	683(63.3)	396(36.7)	
**Educational status**				
No education	2,580(54.1)	1,395(54.1)	1,185(45.9)	X2= 34.29 p<0.001
Primary	1,577(33.0)	882(55.9)	695(44.1)	
Secondary	387(8.1)	242(62.5)	145(37.5)	
Higher	227(4.8)	153(67.5)	74(32.5)	

Family size				
>5	2,258(46.7)	1,248(55.3)	1,010(44.7)	X2= 2.43 p= 0.118
≤5	2,514(53.3)	1,424(56.7)	1,090(43.3)	
**Parity**				
Nulliparous	30(0.7)	18(58.5)	12(41.5)	X2= 0.897 p=0.217
Primiparous	1,193(28.9)	691(58.9)	502(42.1)	
Multiparous	2,241(45.9)	1,228(54.8)	1,014(45.2)	
Grand multiparous	1,307(27.5)	736(56.3)	571(43.7)	
**Frequency of ANC**				
One visit	313(6.6)	107(34.2)	206(65.8)	X2= 191.19 p<0.001
2 visits	581(12.2)	258(44.4)	323(55.6)	
3 visits	1,419(29.7)	766(54.0)	653(46.0)	
≥4 visits	2,458(51.5)	1,541(62.7)	918(37.3)	
**Listen to radio**				
Not at all	3,162(65.5)	1,749(55.3)	1,413(44.7)	X2= 69.13 p<0.001
Less than once a week	793(16.9)	451(56.9)	342(43.1)	
At least once a week	817(17.6)	472(57.7)	345(42.3)	
**Own mobile phone**				
Yes	1,166(26.7)	712(61.1)	454(38.9)	X2= 54.19 p<0.001
No	3,605(73.3)	1,959(54.3)	1,646(45.7)	
**Covered by Health Insurance schemes**				
Yes	241(6.9)	183(76.0)	58(24.0)	X2= 32.91 p<0.001
No	4,530(93.1)	2,488(54.9)	2,042(45.1)	
**Access to money for seeking medical care**				
Big problem	2,560(53.7)	1,354(52.9)	1,207(47.1)	X2= 46.34 p<0.001
Not a big problem	2,211(46.3)	1,318(59.6)	893(40.4)	

The level of Missing BPCR messages

During the 2016 EDHS, 44.0% (95% CI: 42.6, 45.4%) of the surveyed women reported that they missed all BPCR messages during their last pregnancy. The proportion of women who missed the messages was highest in the Afar and Somali regions, with 78.1% and 67.2%, respectively. The top three messages not received by the majority of women were about arranging a potential blood donor (92.7%), identifying a companion during childbirth (90.8%), and securing emergency transportation (84.1%) (Table 3).

Table 3: The level of missing BPCR messages during pregnancy across regions of Ethiopia, EDHS 2016

Regions	Women who missed each BPCR message [Weighted frequency (%)]						
	About place of birth	About supplies needed for birth	About emergency transportation	About emergency fund/money	About companion during childbirth	About potential blood donors	Missing all items
Tigray	154(31.8)	289(59.9)	353(77.8)	392(80.6)	425(87.6)	444(91.4)	111(22.8)
Afar	30(82.4)	33(90.1)	34(95.9)	35(96.8)	36(97.2)	36(97.7)	29(78.1)
Amhara	426(38.6)	845(76.6)	960(88.0)	965(87.4)	998(90.4)	1,005(91.0)	377(34.1)
Oromia	1,010(62.9)	1,172(72.9)	1,372(85.3)	1,353(84.2)	1,497(93.1)	1,521(94.6)	863(53.7)

Somali	81(68.9)	99(83.6)	108(91.2)	107(90.3)	110(93.3)	114(96.2)	79(67.2)
Benishangul	31(56.3)	45(80.4)	48(86.4)	46(83.0)	49(87.3)	51(81.8)	29(51.1)
SNNPR	582(52.3)	764(68.5)	945(84.8)	894(80.2)	1,004(90.1)	1,023(91.8)	511(45.8)
Gambella	9(61.1)	11(75.6)	13(83.8)	13(87.3)	14(93.0)	14(93.0)	9(57.1)
Harari	7(52.6)	11(79.6)	10(78.6)	11(82.9)	12(87.2)	12(88.6)	5(39.2)
Addis Ababa	111(58.0)	103(53.6)	142(74.1)	134(68.8)	163(85.2)	177(92.4)	71(37.1)
Diredawa	18(60.9)	23(79.9)	26(89.6)	24(83.2)	28(94.8)	28(94.4)	16(54.4)
**Total**	2,461(51.6)	3,395(71.2)	4,013(84.1)	3,975(83.3)	4,336(90.8)	4,424(92.7)	2,099(44.0)

Spatial autocorrelation of not receiving BPCR messages

The spatial autocorrelation analysis revealed that there was a considerable spatial variation in not receiving BPCR messages across the country (Global Moran's I value 0.218, p-value<0.001). The clustered patterns (on the right sides) suggest that missed BPCR messages happened at a high rate throughout the study area. The Z-score of 7.212 implies that there is less than a 1% possibility that this clustered pattern is due to random chance (Figure 1).

Fig 1. The global spatial autocorrelation of missing BPCR messages during pregnancy in Ethiopia, EDHS 2016

The hot spot (Getis-Ord Gi*) analysis for missing BPCR messages

Hot spot analysis enables the detection of either high or low statistically significant coverage regions with the absence of BPCR messages. The northern, southern, and western Somali, central Afar, and Gambella regions were found as statistically significant hotspots (red dots) for not getting BPCR messages. While considerable cold spot areas were found in Tigray, Amhara, and Addis Ababa (Figure 2).

Fig 2. Hot spot and Cold spot analysis of missing BPCR messages across regions in Ethiopia, EDHS 2016

Interpolation using GIS mapping

Based on EDHS-2016 sampled data, areas with a relatively high proportion of missing BPCR messages (red) were predicted in Somali, Afar, and some parts of Oromia regions. In contrast, the predicted proportion of high receipt of BPCR messages (green shaded) encompasses the entire Tigray, Western Amhara, and central Addis Ababa regions (Figure 3).

Fig 3. Ordinary Kriging interpolation of the spatial distribution of missing BPCR messages in Ethiopia, EDHS 2016.

Predictors of missing BPCR messages

The global ordinary least square (OLS) analysis result

OLS is a global regression model that uses a single equation to estimate the relationship between the dependent and independent variables, and it assumes the coefficients of each

variable are homogenous across the study area. By meeting all of the fundamental assumptions, the initially employed spatial analysis, OLS model was fitted for the candidate explanatory variables. The model has been tuned to detect multicollinearity among the independent variables, with a mean VIF of less than 10. The adjusted R2 indicated that 24.2% (adjusted R2 = 0.242) of the variation in missing BPCR messages was explained by those six explanatory variables in the model. The robust probabilities for the intercept and all the included explanatory showed coefficient significance ($p < 0.01$) for the explanatory variables. Jarque-Bera statistics with p-values greater than 0.05 suggest that the model prediction using OLS was not biased (the assumption of residual normality was met). The Koenker statistics in the model, on the other hand, exhibited a statistically significant p-value (p=0.0198), indicating that the regression model is inconsistent across the study area (as geographic position changes, so does the association of variables. This implies that the GWR model was deemed more suited for estimating model parameters. Consequently, being in the poorest wealth quintile, having only one ANC visit, having no access to listening to the radio, having difficulty accessing money to get medical care, not having a mobile phone, and not being covered by any health insurance scheme were the spatial determinants of hot spot areas for missing BPCR messages (Table 4).

Table 4: Summary of OLS results for not receiving BPCR messages in Ethiopia, EDHS 2016

Variable	Coefficient	SE	t-Statistic	Probability	Robust SE	Robust t-statistics	Robust probability	VIF
Intercept	-0.045	0.084	-4.21	0.021	0.067	-3.32	<0.001	---
Women in the poorest wealth quintiles	0.233	0.037	6.33	<0.001	0.041	5.65	<0.001	1.39
Women who received only one ANC visit	0.287	0.079	3.62	<0.001	0.070	4.11	<0.001	1.17
Women who never listened to a radio	0.144	0.043	3.32	<0.001	0.048	2.99	0.003	1.47
Women who didn't have a mobile phone	-0.132	0.035	-3.75	<0.001	0.036	-3.60	<0.001	1.26
Women who faced difficulty in getting money	0.134	0.038	3.51	<0.001	0.041	3.26	0.001	1.25
Women who were not covered by any health insurance schemes	0.367	0.085	4.32	<0.001	0.066	5.51	<0.001	1.03

**OLS Diagnostics**			
Number of Observations:	607	Akaike's Information Criterion (AICc)	181.81
Multiple R-Squared	0.252	Adjusted R-Squared [d]:	0. 242
Joint F-Statistic	28.771	Prob(>F), (7,599) degrees of freedom	<0.001
Joint Wald Statistic	253.005	Prob(>chi-squared),(7) degrees of freedom	<0.001
Koenker (BP) Statistic	3.959	Prob(>chi-squared), (7) degrees of freedom	0.0198
Jarque-Bera Statistic	1.214	Prob(>chi-squared), (2) degrees of freedom	0.0604

Geographically weighted regression (GWR)

Although the OLS model assumes that the association between each covariate and the outcome of interest is stationary across the study area, significant Koenker (BP) Statistics ($p<0.01$) show that this assumption is violated. To deal with this breach of the global (OLS) model's stationarity assumption, the local (GWR) model was fitted to provide credible estimates. The GWR analysis showed a considerable improvement over the global model (OLS). The AICc value decreased from 181.81 in the OLS model to 161.21 in the GWR model. Likewise, the adjusted R^2 in OLS ascended to 0.332 in GWR, indicating that the local model improved the capacity for predicting hotspots of missing BPCR messages. When the AICc values of two models (OLS versus GWR) differ by more than three, the model with the lower AICc is considered superior. Overall, GWR analysis outperformed the model developed with OLS in the current study.

The predicted coefficient of being in the poorest wealth quintile varies across the regions, revealing an uneven association between poverty and the proportion of missing BPCR messages. As the proportion of women living in the poorest wealth quintile increased, so did the proportion of missing BPCR messages in most parts of Afar and Somali, as well as in some parts of Gambella and Benishangul Gumuz. In those regions, the predicted coefficient ranges from 0.67 to 1.00 units. The northern part of the country, on the other hand, had the lowest coefficient for women in the poorest wealth quintile (northern, southern, and central Amhara region, and the majority of Tigray region). The red dotted area denotes a strong influence (high predicted coefficient) of the explanatory variables on missing BPCR messages (Figure 4).

Fig 4. Spatial mapping of GWR coefficients of women in the poorest wealth quintile for predicting missing BPCR messages in Ethiopia, EDHS 2016

Besides, receiving the lowest number of ANC visits (only one visit) was associated with the spatial variation of missing BPCR messages. As the proportion of women who received only one ANC visit increased, the percentage of missing BPCR messages among pregnant women in various parts of Afar, western, southern, and northern Somali, and some parts of SNNPR and Diredawa. The weakest relationship was detected in the majority of parts of Tigray, and Amhara regions (Figure 5).

Fig 5. Spatial mapping of GWR coefficients of women with only one ANC visit for predicting missing BPCR messages in Ethiopia, EDHS 2016

The proportion of women who never listen to the radio was found to be strongly associated with an increased risk of missing BPCR messages, with the greatest effect observed in Somalia (southern, northern, and western parts), Afar (eastern and southern parts), and Gambella

(northern and southern parts). The weakest association with the predicted coefficient of 0.04 to 0.32 was found in the northern Tigray and some parts of the Amhara regions (Figure 6).

Fig 6. Spatial mapping of GWR coefficients of women who never listen to the radio for predicting missing BPCR messages in Ethiopia, EDHS 2016.

Furthermore, not having a mobile phone was associated with a regional difference in not getting BPCR messages. Women in the northern, southern, and western parts of the Somali region and the eastern border of Afar who did not have mobile phones had a higher likelihood of missing BPCR messages (Figure 7).

Fig 7. Spatial mapping of GWR coefficients of women who didn't own a mobile phone for predicting missing BPCR messages in Ethiopia, EDHS 2016

The predicted coefficient of difficulty in obtaining money for medical care on the level of missing BPCR messages varies by geographical region. As the proportion of women who faced difficulty in getting money increased, the failure to receive BPCR messages in the northern and southern portions of Somali, as well as the central and south-western parts of the Afar regions. Additionally, it encompasses some parts of Diredawa and Hareri, Oromia (southern), Gambella (northern), and the Benishangul Gumuz (southern) regions (Figure 8).

Fig 8. Spatial mapping of GWR coefficients of women who faced difficulty in getting money for predicting missing BPCR messages in Ethiopia, EDHS 2016

Finally, not covered by any type of health insurance scheme has a positive relationship with missing BPCR messages. As the proportion of women who were not covered by any HI schemes increased, the prevalence of missing BPCR increased greatly in all regions except Amhara and Tigray (Figure 9).

Fig 9. Spatial mapping of GWR coefficients of women who were not covered by health insurance schemes for predicting missing BPCR messages in Ethiopia, EDHS 2016

Discussion

This is the first study in Ethiopia that aimed at looking at the geographical variation of missing BPCR messages during pregnancy and its predictors. The findings of spatial global Moran's analysis found that the percentage of women who did not get BPCR messages varied geographically. The overall prevalence of missing BPCR messages in Ethiopia was found to be 44.0% (95% CI: 42.6, 45.4). As per the analysis, there was a high clustering (hot spot) of not receiving messages in Somalia, Afar, and Gambella regions. Those regions were known for low uptake of maternal and child health services [39-41]. Prior pocket studies conducted in those regions also supported this finding [42-44]. This clustering might be due to a variety of

factors. To begin, those two regions are known to have fewer healthcare facilities, inadequate healthcare providers, and lesser availability of medical supplies and equipment to access maternal health services when compared to other regions[40, 45]. In addition, these regions frequently have remote and difficult-to-reach areas with limited transportation, cultural practices that may take precedence over modern medical care, and lower socioeconomic conditions, such as poverty and a lack of education, making it difficult for pregnant women to access healthcare services on time[40, 41, 46]. Furthermore, some populations in these regions live nomadic or semi-nomadic lives, which might make it difficult for pregnant women to get consistent healthcare services because their locations shift over time [40, 47]. All these can lead to a lack of proper follow-up during pregnancy which might lead to missing BPCR messages.

In the local (GWR) model, being in the poorest wealth quintile, having only one ANC visit, having no access to listening to the radio, having difficulty accessing money to get medical care, not having a mobile phone, and not being covered by any health insurance scheme were identified as significant predictors for the observed clustering (hot spot) in missing BPCR messages in Ethiopia.

Living in a household with the poorest wealth quintile was revealed to be a statistically significant predictor of hot spot for not receiving BPCR messages in most parts of Afar and Somali, as well as in some parts of Gambella and Benishangul Gumuz. In those regions, the predicted coefficient ranges from 0.67 to 1.00 units. This was supported by studies conducted in Nigeria[48], Ghana[49], and Ethiopia[14, 50, 51]. These links may be explained in part by the low utilization of maternal health services in those spots[39-41]. In addition, those women in the poorest wealth quintile are more likely to experience financial barriers in accessing maternal healthcare especially ANC, which is a vital entry point to get information about BPCR messages. In addition, poverty and economic challenges can cause extra pressures on women and families, by hampering access to media and the ability to make decisions[52]. All of this might influence their capacity to seek healthcare services as well as receive key BPCR messages. It is vital to emphasize that being in the poorest wealth quintile might lead to a cycle of inadequate service use and poor health outcomes. Improving the issue necessitates multifaceted approaches that address economic disparities, enhance public infrastructures, promote education, and engage communities in addressing cultural norms and beliefs [53].

Similarly, the frequency of ANC visits is a key spatial predictor of hotspots of missing BPCR messages across the region. As the proportion of women who received only one ANC visit

increased, the percentage of missing BPCR messages in various parts of Afar, western, southern, and northern Somali, and some parts of SNNPR and Diredawa. Studies conducted in Tanzania[54], Egypt[55], Kenya[56], and Ethiopia[43, 51] found that having a low number of ANC visits was positively related to not receiving BPCR messages. This could be attributed to a variety of reasons. To begin, receiving BPCR messages entails a sequence of steps that must be followed to ensure a safe pregnancy and childbirth, and if a woman just attends one visit, she may not obtain comprehensive information about all of these elements. In addition, with changing circumstances, not getting adequate visits resulted in missing out on all of the WHO-recommended BPCR messages. Overall, because pregnancy is a dynamic process, obtaining only one ANC visit can result in missing BPCR messages due to a lack of comprehensive education, support, tailored updates, engagement, and follow-ups that subsequent visits offer[57, 58]. Thus, pregnant women need to attend all directed ANC visits to ensure they obtain the appropriate information and care for a safe pregnancy and childbirth.

Not having access to the radio was found to be strongly associated with an increased risk of missing BPCR messages in Somalia (southern, northern, and western parts), Afar (eastern and southern parts), and Gambella (northern and southern parts). This was supported by studies conducted in Uganda[59], and Ethiopia[10, 60, 61]. As a result of not tuning in to the radio, women may remain unaware of and miss out on services available to them throughout pregnancy and childbirth, becoming alienated or feeling more lonely and less supported during their pregnancy experience[62]. All these could contribute to missing BPCR messages during pregnancy. Subsequently, a lack of radio access can limit women's ability to make informed decisions and contribute to discrepancies in the uptake of key BPCR messages. Hence, a concerted effort is needed to enhance the accessibility of radio, to ensure that no woman is deprived of those key BPCR messages during pregnancy. Similarly, not having a mobile phone was associated with a regional difference in not getting BPCR messages in the northern, southern, and western parts of the Somali region and the eastern border of Afar. Studies conducted elsewhere [63-65] showed that there was a strong association between having a mobile phone and receipt of BPCR messages. This could be because women without mobile phones were less likely to receive health-related information, appointment reminders, health awareness campaigns, and peer support, resulting in BPCR messages being overlooked [66]. Overall, mobile phones provide a versatile and accessible platform for bridging gaps in maternal healthcare service usage through providing information, support, reminders, and access to healthcare providers, particularly in areas with limited healthcare infrastructure[67].

As a result, it is vital to prioritize digital literacy, network coverage, and cost to promote fair access for all women.

Finally, being not covered by health insurance schemes depicts a positive link with missing BPCR messages; as the proportion of women who were uninsured increased, the prevalence of missing BPCR increased significantly in all regions except Amhara and Tigray. Studies conducted in Rwanda [68], and Nigeria[69] showed high uptake of BPCR messages among those women who were insured. This could be because a lack of health insurance coverage can create hurdles to access and continuity of care, as well as engagement with health service delivery points, due to cost concerns and financial hardship[70, 71]. As a result, people may connect with healthcare providers less frequently, missing out on vital information about BPCR messages. On the other hand, women who were covered by health insurance were more likely to visit health facilities, and this trend made them more familiar with the healthcare system, which in turn boosted their health-seeking behavior during pregnancy[72].

The current study has both strengths and limitations.

The findings of this study were not without limitations. The study has the following strengths. First, the findings from the current study were based on an analysis of nationally representative data collected through standardized and validated data collection tools and procedures, making the findings more generalizable. In addition, Hot Spot and geographically weighted regression analyses were carried out, and the findings can help government and program planners design geographically focused public health interventions on the identified predictors to improve BPCR message uptake. Despite the strengths, the findings should be interpreted considering the following limitations. Firstly, to ensure the privacy of respondents or the community, the geographical coordinates of clusters were displaced by up to 2km in urban areas, 5km for most clusters in rural areas, and 10km for 1% of clusters in rural areas; this may impact estimated cluster effects in the spatial regression. Secondly, because the data were collected through retrospective interviews of women who gave birth within five years of the survey, the findings may be subject to recall bias. Finally, due to the cross-sectional nature of the data, the findings are likely to suffer from social desirability bias and fail to address the cause-effect relationship.

Conclusion

The level of missing BPCR messages during pregnancy was found to be high in Ethiopia with a significant spatial variation across regions. Statistically significant hotspot areas for not receiving BPCR messages during pregnancy were northern, southern, and western Somali, central Afar, and Gambella regions. Living in the poorest wealth quintile, receiving only one

ANC visit, not having access to listening to the radio, having difficulty accessing money to get medical care, not having a mobile phone, and being not covered by health insurance were identified as significant predictors of hot spots for missing BPCR messages. Hence, the government and policymakers should tailor strategies for hotspot areas by implementing activities and interventions that increase women's economic capability, health-seeking behavior during pregnancy, and exposure to media by easing access to radio and mobile phones. Furthermore, local governments should focus on strategies that promote universal health coverage through enrolling citizens in health insurance schemes.

Abbreviations

AIC: Akaike's information criterion; ANC: Antenatal Care; BPCR: Birth preparedness and complication readiness; CSA: Central Statistical Agency; EDHS: Ethiopian Demographic and Health Survey; GWR: Geographically Weighted Regression; OLS: Ordinary Least Square; WHO: World Health Organization

Acknowledgment

We are grateful to ICF macro (Calverton, USA) for providing the 2016 DHS data of Ethiopia.

Funding:

There is no funding to report

Data Availability

The data for this study were obtained from the DHS program with a reasonable request. Thus, the one who needs the data supporting the findings of this study can get it in anonymized form from the DHS website at https://www.dhsprogram.com upon reasonable request in the same manner as the authors did.

Disclosure

We would like to assure you that, with full responsibility and communication, the document has been submitted. We indicated that there is no conflict of interest with other individuals or organizations that could influence or bias the content of the paper inappropriately.

Supporting information:

S1File: Output for incremental spatial autocorrelation

References

1. *WHO, Maternal mortality,Key facts, 2023 https://www.who.int/news-room/fact-sheets/detail/maternal-mortality.*
2. Organization, W.H., *Trends in maternal mortality 2000 to 2020: estimates by WHO, UNICEF, UNFPA, World Bank Group and UNDESA/Population Division: executive summary.* 2023.
3. Zarocostas, J., *Global maternal mortality rates stagnating.* The Lancet, 2023. **401**(10377): p. 632.

4. *USAID, Ethiopia, Maternal, Neonatal and Child Health https://www.usaid.gov/ethiopia/global-health/maternal-neonatal-and-child-health#:~:text=Since%202000%2C%20Ethiopia%20has%20reduced,1%2C000%20are%20still%20too%20high.*
5. CSA, I., *Central Statistical Agency (CSA)[Ethiopia] and ICF. Ethiopia Demographic and Health Survey 2016. Addis Ababa, Ethiopia, and Rockville, Maryland, USA: CSA and ICF; 2016*. 2017.
6. Solnes Miltenburg, A., et al., *Impact of birth preparedness and complication readiness interventions on birth with a skilled attendant: a systematic review.* PloS one, 2015. **10**(11): p. e0143382.
7. Organization, W.H., *Birth and emergency preparedness in antenatal care.* Standards for Maternal and Neonatal Care. Geneva, World Health Organization, 2006: p. 1-6.
8. Jhpiego, *Monitoring birth preparedness and complication readiness: tools and indicators for maternal and newborn health*. 2004.
9. Sk, M.I.K., et al., *Praying until death: revisiting three delays model to contextualize the socio-cultural factors associated with maternal deaths in a region with high prevalence of eclampsia in India.* BMC pregnancy and childbirth, 2019. **19**: p. 1-11.
10. Habte, A., A. Tamene, and D. Woldeyohannes, *The uptake of WHO-recommended birth preparedness and complication readiness messages during pregnancy and its determinants among Ethiopian women: A multilevel mixed-effect analyses of 2016 demographic health survey.* Plos one, 2023. **18**(3): p. e0282792.
11. Ohenhen, V., et al., *Birth preparedness and complication readiness: Evaluating the "know-do" gap among women receiving antenatal care in Benin City, Nigeria.* Journal of Biosocial Science, 2023: p. 1-15.
12. Ketema, D.B., et al., *Effects of maternal education on birth preparedness and complication readiness among Ethiopian pregnant women: a systematic review and meta-analysis.* BMC pregnancy and childbirth, 2020. **20**(1): p. 1-9.
13. Markos, D. and D. Bogale, *Birth preparedness and complication readiness among women of child bearing age group in Goba woreda, Oromia region, Ethiopia.* BMC pregnancy and childbirth, 2014. **14**: p. 1-9.
14. Debelew, G.T., M.F. Afework, and A.W. Yalew, *Factors affecting birth preparedness and complication readiness in Jimma Zone, Southwest Ethiopia: a multilevel analysis.* Pan African Medical Journal, 2014. **19**(1).
15. Bitew, Y., W. Awoke, and S. Chekol, *Birth preparedness and complication readiness practice and associated factors among pregnant women, Northwest Ethiopia.* International scholarly research notices, 2016. **2016**.
16. Kaso, M. and M. Addisse, *Birth preparedness and complication readiness in Robe Woreda, Arsi Zone, Oromia Region, Central Ethiopia: a cross-sectional study.* Reproductive health, 2014. **11**: p. 1-12.
17. Worku, M., et al., *Male involvement and associated factors in birth preparedness and complication readiness in Debre Berhan town, North East Ethiopia.* The Pan African Medical Journal, 2020. **35**.
18. Debelie, T.Z., et al., *Birth preparedness and complication readiness practice and associated factors among pregnant women in Northwest Ethiopia: 2018.* PLOS ONE, 2021. **16**(4): p. e0249083.
19. *ICF International. 2013. Incorporating Geographic Information into Demographic and Health Surveys: A Field Guide to GPS Data Collection. Calverton, Maryland, USA: ICF International https://dhsprogram.com/pubs/pdf/DHSM9/DHS_GPS_Manual_English_A4_24May2013_DHSM9.pdf.*
20. Jaleta, A.D., et al., *Birth Preparedness, Complication Readiness, and Determinants Among Women Attending Antenatal Care from Ethiopia: A Cross-sectional Facility-Based Survey.* European Journal of Clinical and Biomedical Sciences, 2021. **7**(3): p. 37-43.

21. Begashaw, B., et al., *Assessment of birth preparedness and complication readiness among pregnant mothers attending ante Natal Care Service in Mizan-Tepi University Teaching Hospital, South West Ethiopia.* Clinics Mother Child Health, 2017. **14**.
22. Kassahun, A. and A. Zewdie, *Decision-making autonomy in maternal health service use and associated factors among women in Mettu District, Southwest Ethiopia: a community-based cross-sectional study.* BMJ open, 2022. **12**(5): p. e059307.
23. Asaolu, I.O., et al., *Measuring women's Empowerment in sub-Saharan Africa: exploratory and confirmatory factor analyses of the demographic and health surveys.* Frontiers in psychology, 2018. **9**: p. 994.
24. Chen, Y., *An analytical process of spatial autocorrelation functions based on Moran's index.* PLoS One, 2021. **16**(4): p. e0249589.
25. Ord, J.K. and A. Getis, *Local spatial autocorrelation statistics: distributional issues and an application.* Geographical analysis, 1995. **27**(4): p. 286-306.
26. Lu, J., *Incremental autocorrelation calculation for streamed data using components*. 2019, Google Patents.
27. Chen, Y., *New approaches for calculating Moran's index of spatial autocorrelation.* PloS one, 2013. **8**(7): p. e68336.
28. Melaku, M.S., et al., *Geographical variation and predictors of zero utilization for a standard maternal continuum of care among women in Ethiopia: a spatial and geographically weighted regression analysis.* BMC Pregnancy and Childbirth, 2022. **22**(1): p. 76.
29. Wulder, M. and B. Boots, *Local spatial autocorrelation characteristics of remotely sensed imagery assessed with the Getis statistic.* International Journal of Remote Sensing, 1998. **19**(11): p. 2223-2231.
30. Getis, A. and J.K. Ord, *The analysis of spatial association by use of distance statistics.* Geographical analysis, 1992. **24**(3): p. 189-206.
31. Meng, J., *Raster data projection transformation based-on Kriging interpolation approximate grid algorithm.* Alexandria Engineering Journal, 2021. **60**(2): p. 2013-2019.
32. Van Beers, W.C. and J.P. Kleijnen. *Kriging interpolation in simulation: a survey*. in *Proceedings of the 2004 Winter Simulation Conference, 2004.* 2004. IEEE.
33. Fortin, M.-J., et al., *Spatial statistics, spatial regression, and graph theory in ecology.* Spatial Statistics, 2012. **1**: p. 100-109.
34. Chi, G. and J. Zhu, *Spatial regression models for demographic analysis.* Population Research and Policy Review, 2008. **27**: p. 17-42.
35. Shrestha, P.M., *Comparison of ordinary least square regression, spatial autoregression, and geographically weighted regression for modeling forest structural attributes using a Geographical Information System (GIS).* Remote Sensing (RS) Approach. Thesis. Canada: University of Calgary. http://people. ucalgary. ca/~ mcdermid/Docs/Theses/Shrestha_2006. pdf (Accessed October 30, 2012), 2006.
36. Beale, C.M., et al., *Regression analysis of spatial data.* Ecology letters, 2010. **13**(2): p. 246-264.
37. Fotheringham, A.S., C. Brunsdon, and M. Charlton, *Geographically weighted regression: the analysis of spatially varying relationships*. 2003: John Wiley & Sons.
38. Chi, G. and D. Wang, *Small-area population forecasting: A geographically weighted regression approach.* The frontiers of applied demography, 2017: p. 449-471.
39. Bobo, F.T., E.A. Yesuf, and M. Woldie, *Inequities in utilization of reproductive and maternal health services in Ethiopia.* International journal for equity in health, 2017. **16**(1): p. 1-8.
40. Wester, K.C., et al., *Best practices for addressing socio-cultural barriers to reproductive, maternal and neonatal health service utilization among women from pastoralist communities of Afar, Ethiopia.* The Ethiopian Journal of Health Development, 2018. **32**(Special Is).
41. Muchie, K.F., *Quality of antenatal care services and completion of four or more antenatal care visits in Ethiopia: a finding based on a demographic and health survey.* BMC pregnancy and childbirth, 2017. **17**(1): p. 1-7.

42. Abebaw, N., et al., *Assessments of Birth Preparedness and Complication Readiness Among Women of Childbirth in Samara Logia Town, Afar, North East Ethiopia.* American Journal of Laboratory Medicine, 2020. **5**(4): p. 95-101.
43. Musa, A. and A. Amano, *Determinants of birth preparedness and complication readiness among pregnant woman attending antenatal care at Dilchora Referral Hospital, Dire Dawa City, East Ethiopia.* Gynecol Obstet (Sunnyvale), 2016. **6**(2): p. 356.
44. Regesu, A.H., et al., *Birth Preparedness and Complication Readiness and Associated Factors among Mothers Attending Antenatal Care at Chiro District Health Centers, West Hararge, Ethiopia.* International Journal of Health Sciences and Research, 2019. **9**(4): p. 187-95.
45. Eba, K., et al., *Mobile health service as an alternative modality for hard-to-reach pastoralist communities of Afar and Somali regions in Ethiopia.* Pastoralism, 2023. **13**(1): p. 1-12.
46. Birch, I., *Barriers affecting demand for reproductive, maternal, newborn and child health (RMNCH) services in Ethiopia.* 2020.
47. Zepro, N.B., et al., *Lived Experiences and Perceptions of Childbirth among Pastoralist Women in North-Eastern Ethiopia: A Multimethod Qualitative Analysis to the WHO Health Systems Responsiveness Framework.* International Journal of Environmental Research and Public Health, 2021. **18**(23): p. 12518.
48. Idowu, A., et al., *Birth preparedness and complication readiness among women attending antenatal clinics in Ogbomoso, South West, Nigeria.* International Journal of Maternal and Child Health and AIDS (IJMA), 2015. **4**(1): p. 47-56.
49. Saaka, M. and L. Alhassan, *Prevalence and predictors of birth preparedness and complication readiness in the Kassena-Nankana district of Ghana: an analytical cross-sectional study.* BMJ open, 2021. **11**(3): p. e042906.
50. Gurara, M.K., et al., *Maternity waiting homes as component of birth preparedness and complication readiness for rural women in hard-to-reach areas in Ethiopia.* Reproductive health, 2021. **18**(1): p. 1-14.
51. Girma, D., A. Waleligne, and H. Dejene, *Birth preparedness and complication readiness practice and associated factors among pregnant women in Central Ethiopia, 2021: A cross-sectional study.* Plos one, 2022. **17**(10): p. e0276496.
52. Wuneh, A.D., et al., *Wealth-based equity in maternal, neonatal, and child health services utilization: a cross-sectional study from Ethiopia.* International Journal for Equity in Health, 2019. **18**: p. 1-9.
53. Mwiti, F. and C. Goulding, *Strategies for community improvement to tackle poverty and gender issues: An ethnography of community based organizations ('Chamas') and women's interventions in the Nairobi slums.* European Journal of Operational Research, 2018. **268**(3): p. 875-886.
54. Urassa, D.P., A.B. Pembe, and F. Mganga, *Birth preparedness and complication readiness among women in Mpwapwa district, Tanzania.* Tanzania journal of health research, 2012. **14**(1).
55. Aziz, M.M., R.M.S. El-Deen, and M.A. Allithy, *Birth preparedness and complication readiness among antenatal care clients in Upper Egypt.* Sexual & Reproductive Healthcare, 2020. **24**: p. 100506.
56. Omari, P.K., Y.A. Afrane, and P. Ouma, *Birth preparedness and complication readiness among women attending antenatal care clinic in health facilities within Bureti Sub County of Kericho County, Kenya.* 2016.
57. Villar, J., et al., *WHO antenatal care randomised trial for the evaluation of a new model of routine antenatal care.* Lancet, 2001. **357**.
58. Organization, W.H., *WHO recommendations on antenatal care for a positive pregnancy experience*. 2016: World Health Organization.

59. Asp, G., et al., *Associations between mass media exposure and birth preparedness among women in southwestern Uganda: a community-based survey.* Global health action, 2014. **7**(1): p. 22904.
60. Andarge, E., A. Nigussie, and M. Wondafrash, *Factors associated with birth preparedness and complication readiness in Southern Ethiopia: a community based cross-sectional study.* BMC pregnancy and childbirth, 2017. **17**(1): p. 1-13.
61. Gedefa, A.G., et al., *Barriers to birth preparedness and complication readiness among pregnant women in rural Ethiopia: using a mixed study design, 2020.* BMJ open, 2023. **13**(4): p. e069565.
62. Fatema, K. and J.T. Lariscy, *Mass media exposure and maternal healthcare utilization in South Asia.* SSM-Population Health, 2020. **11**: p. 100614.
63. Masoi, T.J. and S.M. Kibusi, *Improving pregnant women's knowledge on danger signs and birth preparedness practices using an interactive mobile messaging alert system in Dodoma region, Tanzania: a controlled quasi experimental study.* Reproductive health, 2019. **16**: p. 1-10.
64. Santoso, H.Y.D., et al., *Android application model of "suami siaga plus" as an innovation in birth preparedness and complication readiness (BP/CR) intervention.* Journal of family & reproductive health, 2017. **11**(1): p. 30.
65. Cheptum, J., G. Omoni, and W. Mirie, *Effectiveness of mobile phone text message reminder on birth preparedness in a rural community in Kenya.* Tropical Journal of Obstetrics and Gynaecology, 2019. **36**(1): p. 49-53.
66. Guy, R., et al., *How effective are short message service reminders at increasing clinic attendance? A meta-analysis and systematic review.* Health services research, 2012. **47**(2): p. 614-632.
67. Choudhury, A., O. Asan, and M.M. Choudhury, *Mobile health technology to improve maternal health awareness in tribal populations: mobile for mothers.* Journal of the American Medical Informatics Association, 2021. **28**(11): p. 2467-2474.
68. Smeele, P., et al., *Birth preparedness and complication readiness among pregnant women admitted in a rural hospital in Rwanda.* BMC pregnancy and childbirth, 2018. **18**: p. 1-7.
69. Ekabua, J.E., et al., *Awareness of birth preparedness and complication readiness in southeastern Nigeria.* International Scholarly Research Notices, 2011. **2011**.
70. Kesuma, Z.M. and V. Chongsuvivatwong, *Utilization of the Local Government Health Insurance Scheme (JKA) for maternal health services among women living in underdeveloped areas of Aceh Province, Indonesia.* Asia Pacific Journal of Public Health, 2015. **27**(3): p. 348-359.
71. Habte, A., et al., *Towards universal health coverage: The level and determinants of enrollment in the Community-Based Health Insurance (CBHI) scheme in Ethiopia: A systematic review and meta-analysis.* PloS one, 2022. **17**(8): p. e0272959.
72. Seid, A. and M. Ahmed, *Association between health insurance enrolment and maternal health care service utilization among women in Ethiopia.* BMC Public Health, 2021. **21**(1): p. 1-10.

Click here to access/download;Figure;fig 1.tif

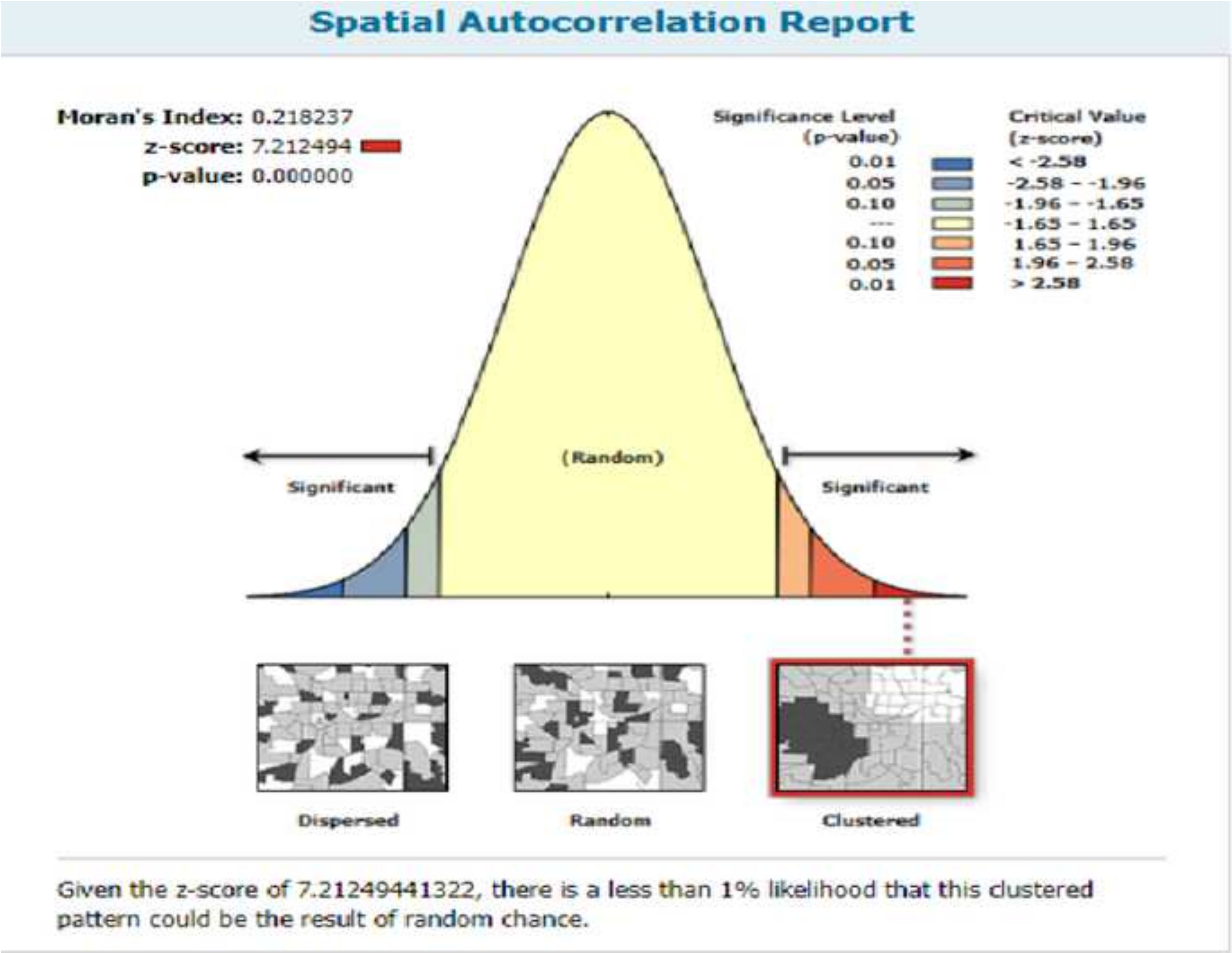

Click here to access/download;Figure;Fig 2.tif

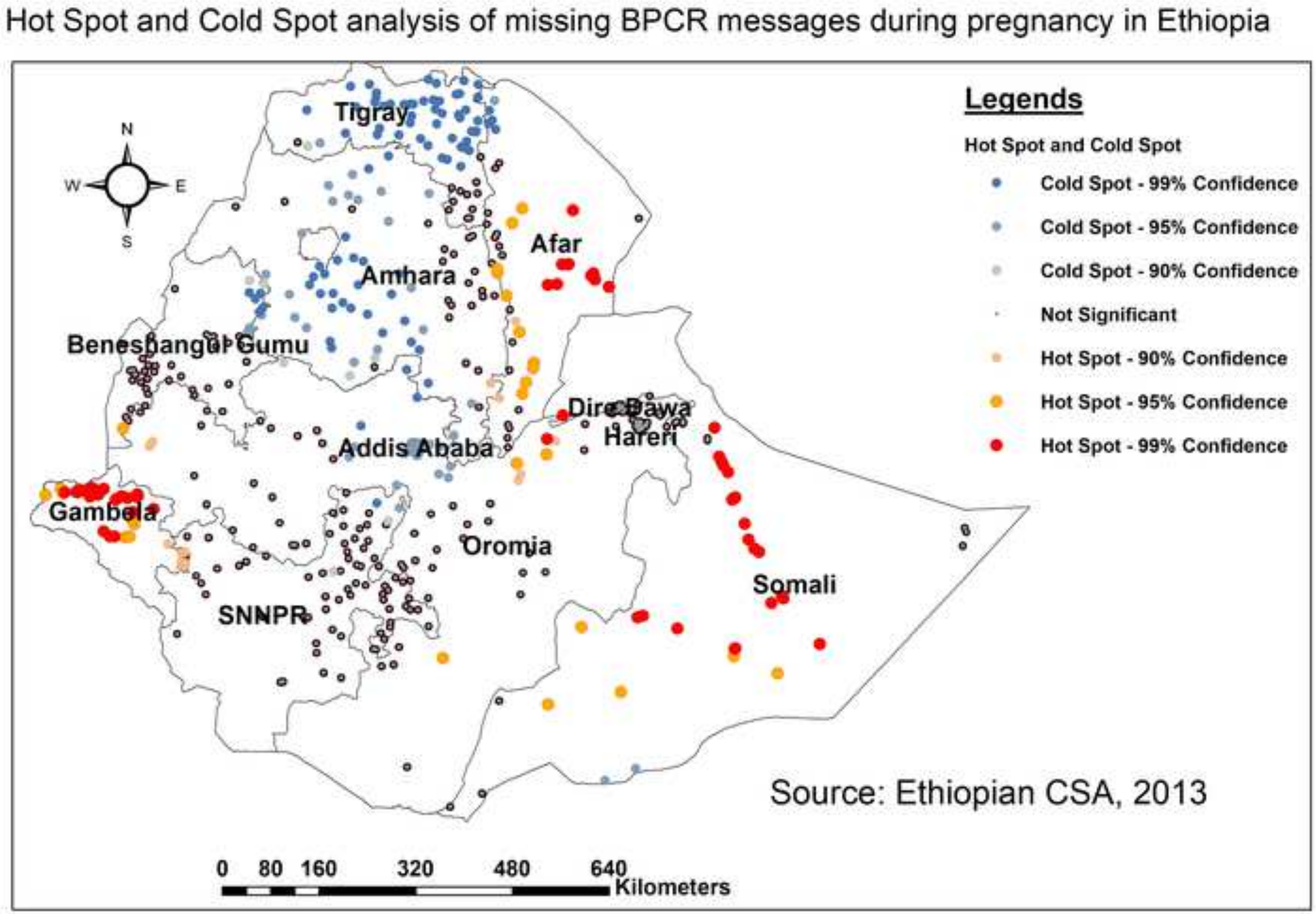

Click here to access/download;Figure;Fig 3.tif

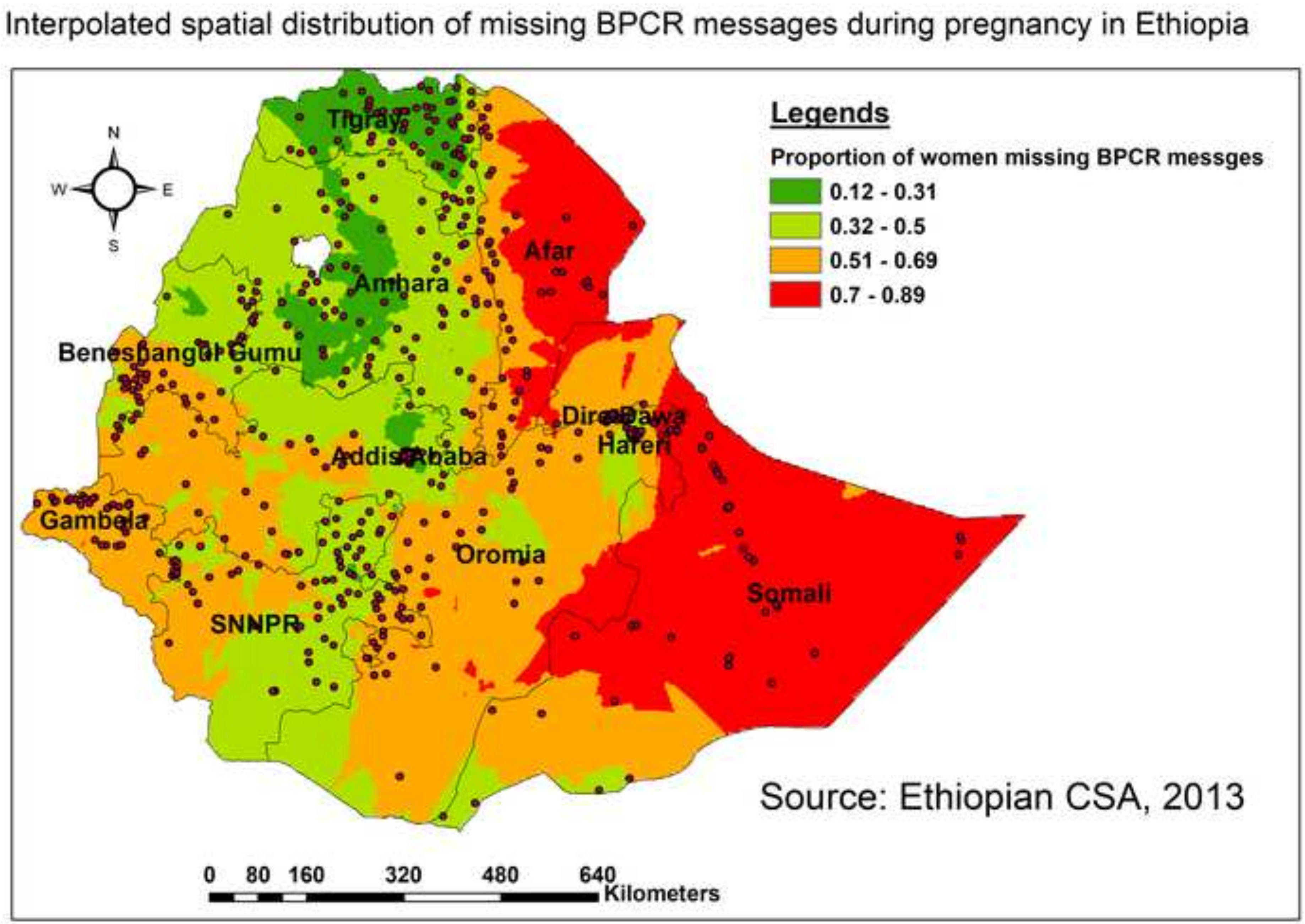

Click here to access/download;Figure;Fig 4.tif

Proportion of women living in the poorest wealth quintile

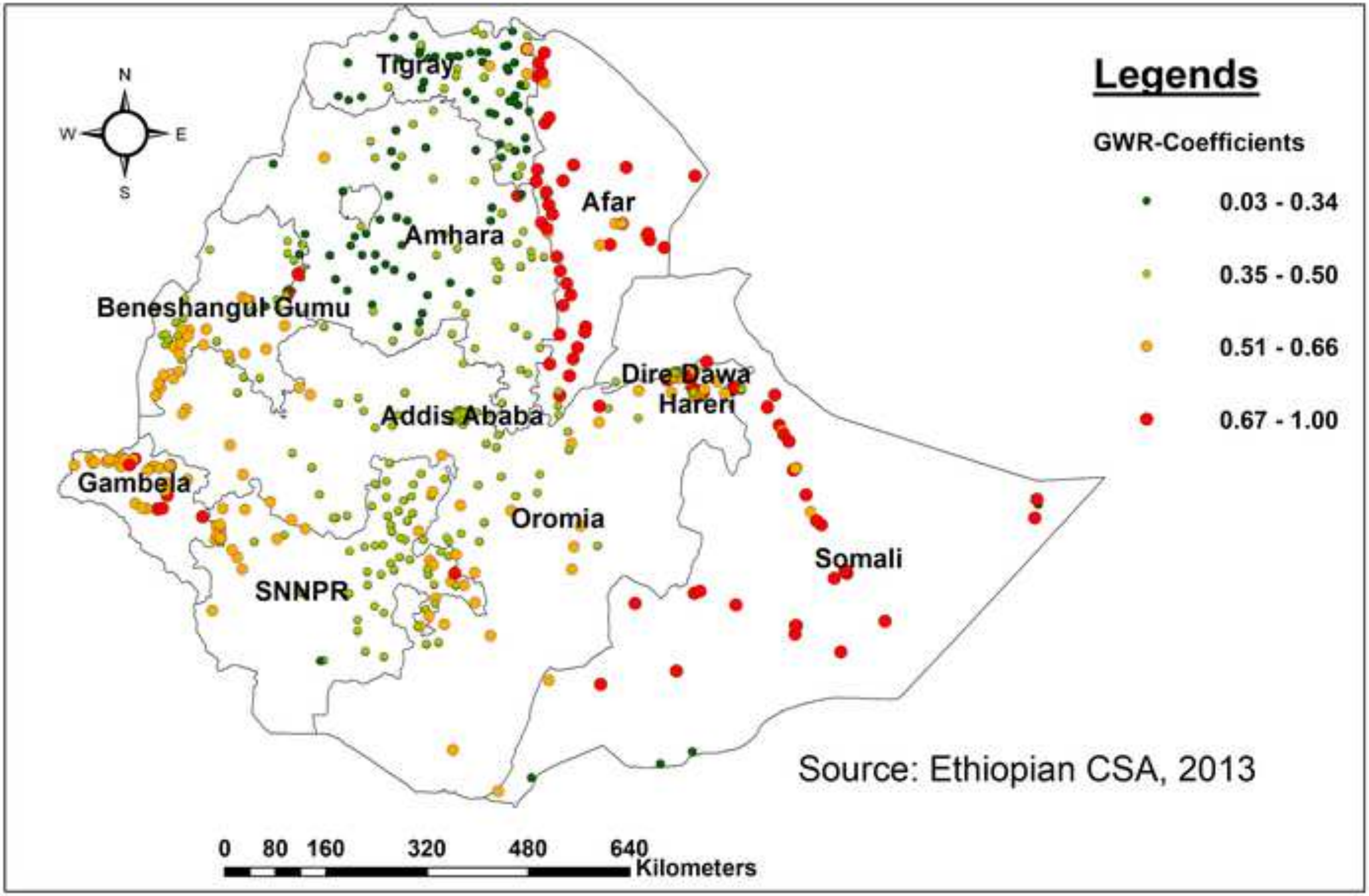

Figure

Click here to access/download;Figure;fig 5.tif

Proportion of women who received only one ANC visit

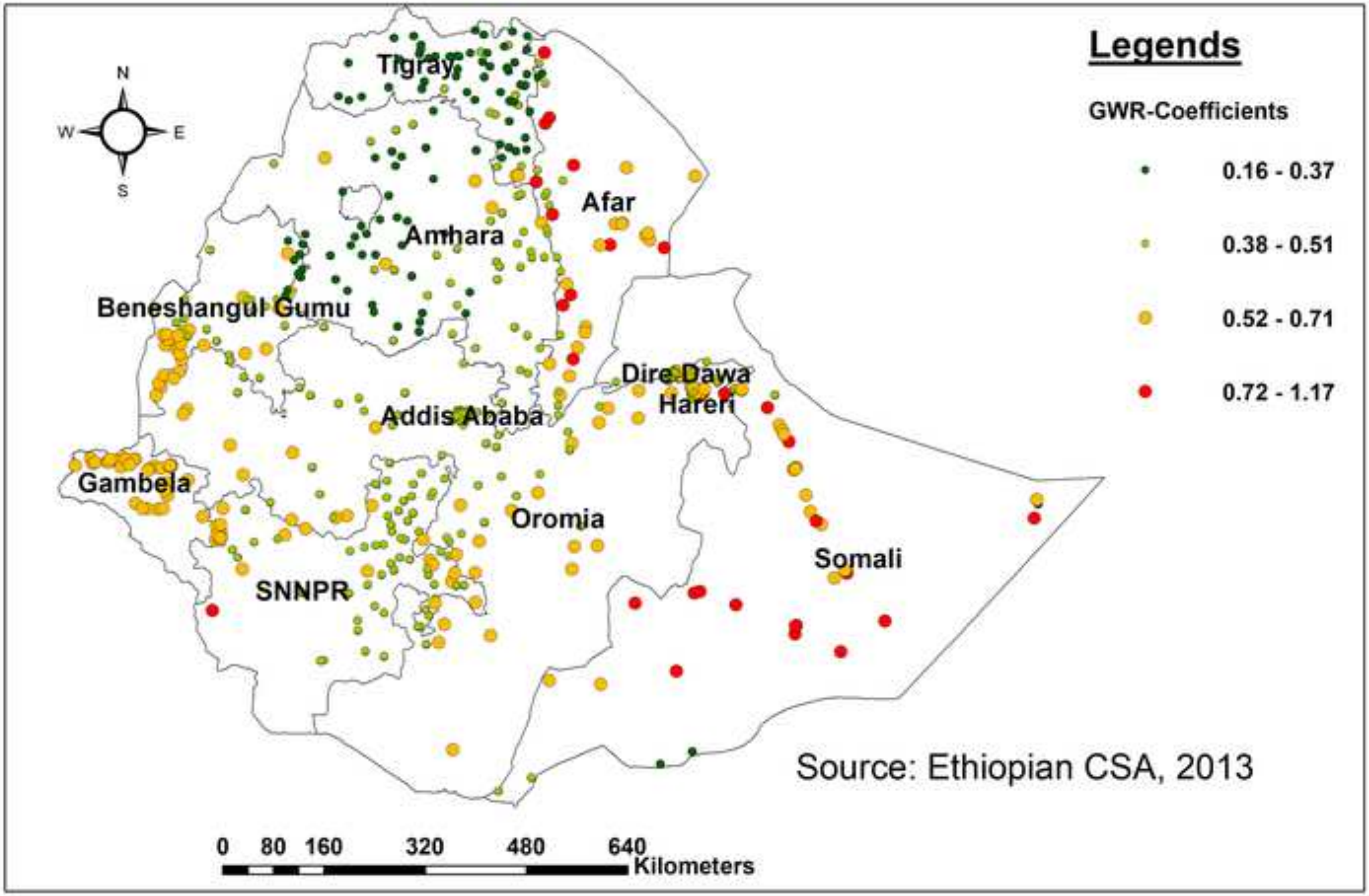

Click here to access/download;Figure;Fig 6.tif

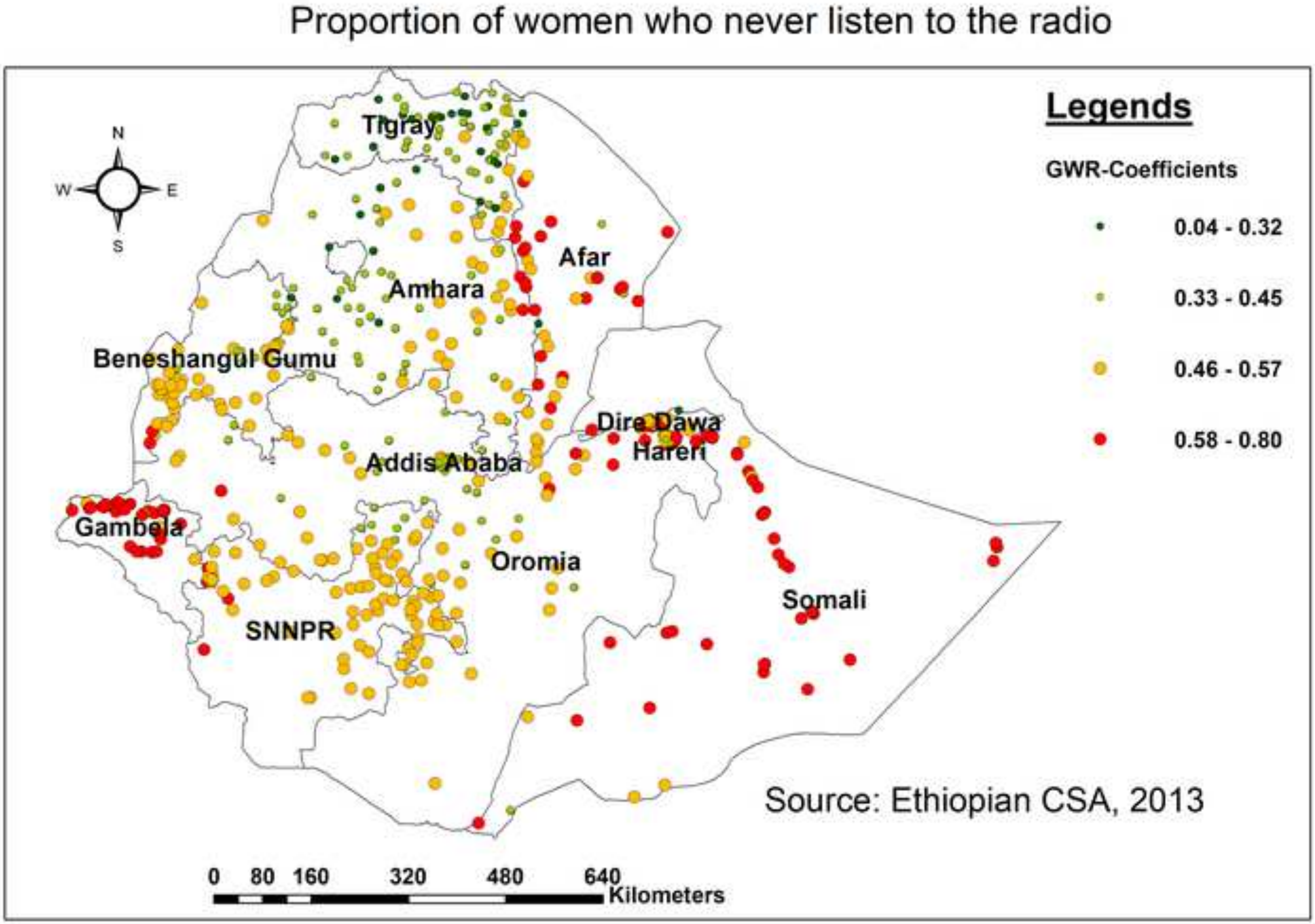

Click here to access/download;Figure;Fig 7.tif

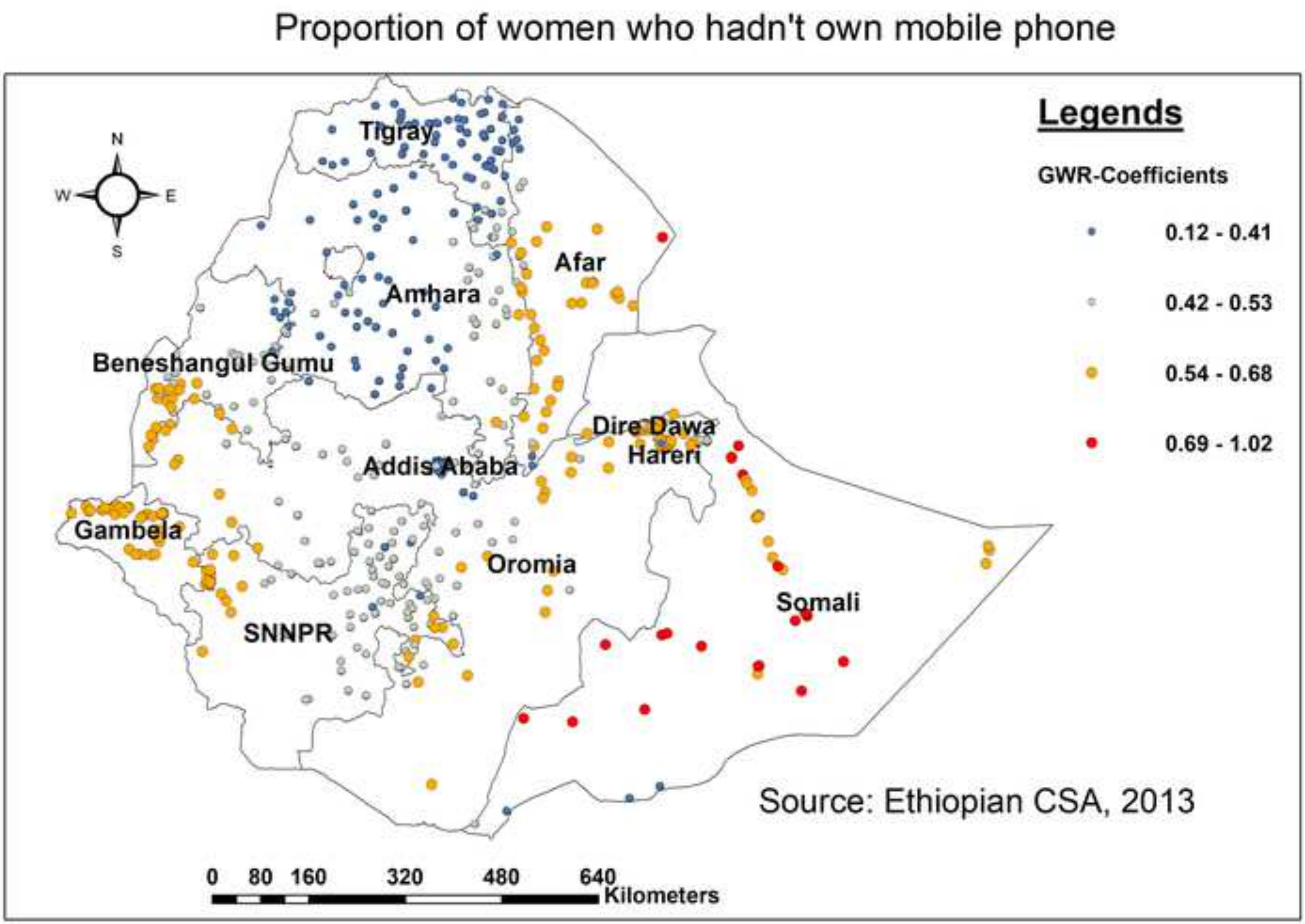

Figure

Click here to access/download;Figure;Fig 8.tif

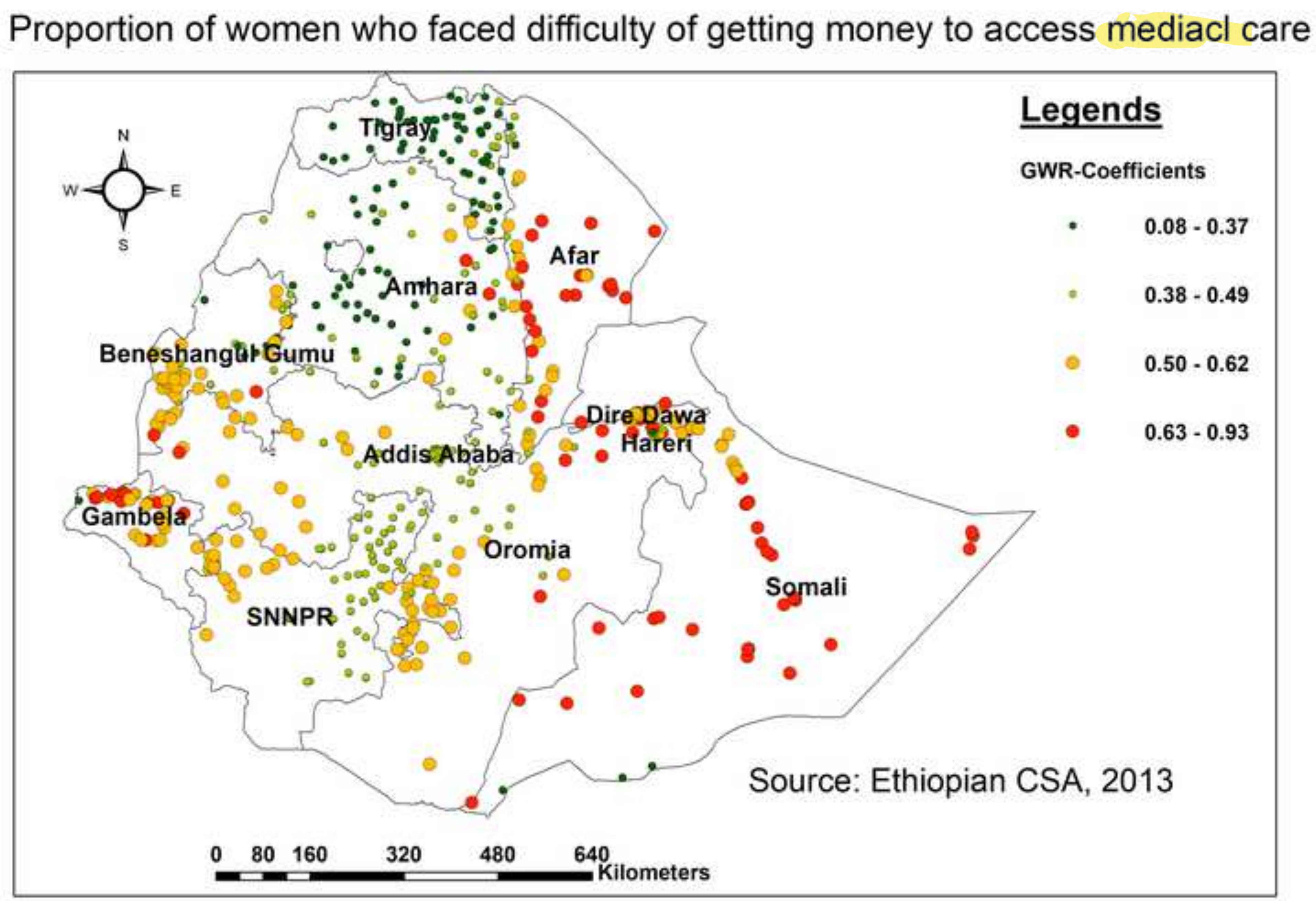

Figure

Click here to access/download;Figure;Fig 9.tif

Proportion of women who were not covered by any Health Insurance scehemes

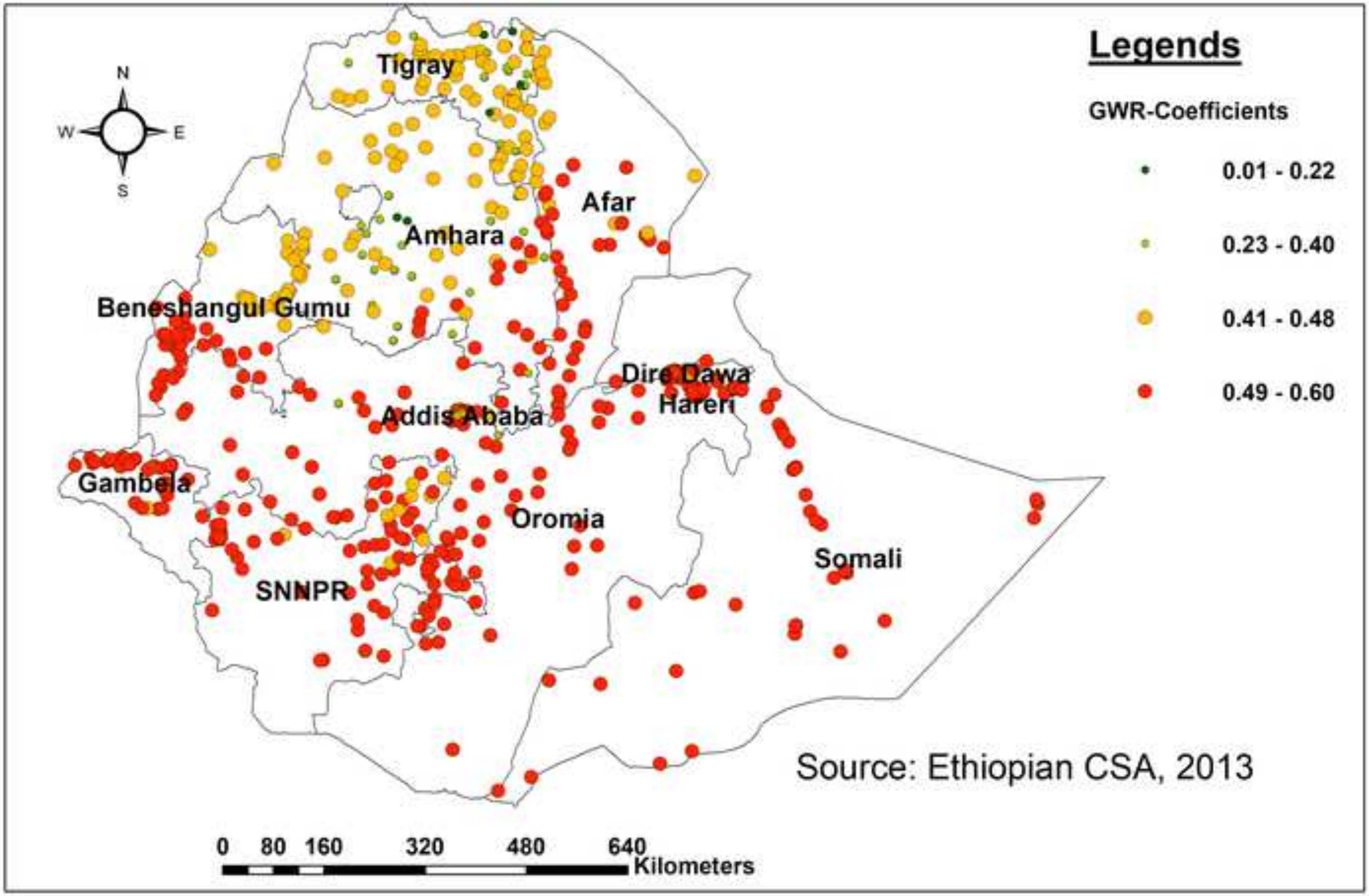

Click here to access/download
Supporting Information
S1File.pdf

Other

Click here to access/download
Supporting Information
STROBE-checklist-v4-combined-PlosMedicine.docx
